# Novel derivatives of brincidofovir and (S)-9-(3-hydroxy-2-phosphonylmethoxypropyl)adenine inhibit orthopoxviruses and human adenoviruses more potently than brincidofovir

**DOI:** 10.1038/s41392-025-02207-w

**Published:** 2025-04-11

**Authors:** Yifan Zhang, Yanmin Wan, Cuiyuan Guo, Zhaoqin Zhu, Chao Qiu, Jiasheng Lu, Yanan Zhou, Jiaojiao Zheng, Fahui Dai, Xiaoyang Cheng, Kunlu Deng, Wanhai Wang, Youchun Wang, Wenhong Zhang

**Affiliations:** 1 Department of Infectious Diseases, Shanghai Key Laboratory of Infectious Diseases and Biosafety Emergency Response, National Medical Center for Infectious Diseases, Huashan Hospital, State Key Laboratory of Genetic Engineering, School of Life Science, Fudan University; Shanghai Sci-Tech Inno Center for Infection & Immunity, Shanghai, China; 2 Department of laboratory medicine, Shanghai Public Health Clinical Center, Shanghai, China; 3 School of Life Sciences, Fudan University, Shanghai, China; 4 Department of radiology, Shanghai Public Health Clinical Center, Shanghai, China; 5 Clinical Laboratory, The First Affiliated Hospital of Zhengzhou University, Key Laboratory of Laboratory Medicine of Henan Province, Zhengzhou, China; 6 Biosafety Level 3 Laboratory, Shanghai Public Health Clinical Center, Shanghai, China; 7 Institutes of biomedical sciences & Shanghai Key Laboratory of Medical Epigenetics, Fudan University, Shanghai, China; 8 Shenzhen Institutes of Advanced Technology, Chinese Academy of Sciences, Shenzhen, China; 9 Risen (Shanghai) Pharma Tech Co. Ltd., Shanghai, China; 10 Institute of Medical Biology, Chinese Academy of Medical Sciences and Peking Union Medical College, Kunming, China; 11 Key Laboratory of Pathogen Infection Prevention and Control (Peking Union Medical College), Ministry of Education; State Key Laboratory of Respiratory Health and Multimorbidity, Beijing, China

**Keywords:** Drug discovery, Drug development

## Abstract

Brincidofovir (BCV) and tecovirimat are the only two chemical drugs that have been approved to treat smallpox and can be requested for monkeypox (Mpox) treatment through a single-patient Emergency Investigational New Drug (EIND) application. Disappointedly, the efficacy of tecovirimat manifested in recent clinical trials is far from being satisfactory, while the clinical efficacy of BCV is still inconclusive. Given that monkeypox virus (MPXV), variola and other emerging orthopoxviruses are posing serious threats to global health, it is urgent to develop better therapeutics. In this study, we tested the antiviral effects of three novel prodrugs, which were designed based on previously reported parent drugs, either (S)-1-(3-hydroxy-2-phosphonylmethoxypropyl)cytosine ((S)-HPMPC, cidofovir) or (S)-9-(3-hydroxy-2-phosphonylmethoxypropyl)adenine ((S)-HPMPA). We found that one of the (S)-HPMPA-based prodrugs, ODE-(S)-HPMPA formate, exhibited significantly better anti-orthopoxvirus activity than BCV both in vitro and in vivo, which also inhibited human adenovirus type 2 and type 21 more efficiently than BCV. Most strikingly, the EC_50_ and EC_90_ of ODE-(S)-HPMPA formate against MPXV were more than 40-fold lower than those of BCV. In contrast, we observed that the anti-herpes simplex virus type 1 (HSV-1) activities of the (S)-HPMPA-based prodrugs were less effective than those of the cidofovir-based prodrugs (BCV and BCV formate), especially in vivo. Moreover, we showed for the first time that cytidine and adenine analog combined therapies could provide mice with complete protection against lethal challenges of both vaccinia and HSV-1. Collectively, we propose that both the ODE-(S)-HPMPA formate and the BCV/ODE-(S)-HPMPA formate combination are worth further investigations for their potential clinical applications.

## Introduction

The genus of Orthopoxvirus comprises 4 species that are pathogenic for human, including variola, vaccinia, cowpox and monkeypox virus (MPXV),^1^ of which variola and MPXV can cause life-threatening diseases. Smallpox was eradicated through massive vaccination, while the incidence of Mpox started to increase after the cessation of routine smallpox vaccination. MPXV clade II caused a global outbreak in 2022 and subsequently, the upsurge of MPXV clade I was declared a public health emergency of international concern in 2024. Preventive and therapeutic counter measures are urgently needed to combat this virus as well as to defend against potential terroristic use of variola.^2–4^

Viral polymerases are the most sought-after targets for developing antiviral drugs.^5–7^ Few dozens of viral RNA or DNA dependent polymerase inhibitors have been licensed for the treatment of human viral diseases, including brincidofovir (BCV), a prodrug of cidofovir (CDV), which is the second FDA approved drug for the treatment of smallpox infection.^7,8^ Given that variola and MPXV are phylogenetically similar, the U.S. CDC suggests that BCV can be requested for the treatment of human Mpox disease in adults and pediatric patients through single-patient FDA emergency use Investigational New Drug (e-IND) for Mpox disease.

BCV is a lipid conjugate of the nucleotide analogue cidofovir, which can inhibit DNA polymerase and terminate chain elongation of many DNA viruses.^9,10^ It has broad antiviral activity against DNA viruses and has been investigated for the prevention and treatment of cytomegalovirus, polyoma viruses, adenoviruses, herpes viruses and orthopoxviruses.^10,11^ Data of previous studies suggested that the in vitro 50% inhibitory concentration (IC_50_) of BCV against MPXV was relatively higher than that of tecovirimat,^12–14^ a specific inhibitor of orthopoxviruses targeting viral p37 protein orthologs.^15^ The p37 protein of orthopoxviruses is critical for the formation of extracellular enveloped virions.^16^ Tecovirimat binds p37 in the region of the H(N)KD phospholipase domain^17^ and blocks the final steps in virus maturation and release from infected cells.^18^ Both BCV and tecovirimat have been used to treat human Mpox disease,^14,19^ but BCV can elevate liver transaminases.^20–22^ Tecovirimat has a relatively better safety profile,^23^ however it has a low barrier to viral resistance. A single amino acid mutation (G277C) to the orthopoxviral p37 protein can lead to resistance to tecovirimat.^16,24^ Resistance to tecovirimat may be especially prone to occurrence among severely immunocompromised Mpox patients under multiple courses of tecovirimat treatment.^25,26^ Besides, according to data released from a clinical trial in the Democratic Republic of the Congo, tecovirimat neither accelerated recovery nor reduced mortality compared to placebo.^27^ In December 2024, NIH announced to discontinue the study of tecovirimat for Mpox (STOMP) because interim results show tecovirimat is safe but does not significantly accelerate lesion resolution or reduce pain.

Mutations associated with CDV resistance have also been identified in multiple orthopoxviruses^14,28^ and human cytomegalovirus.^29^ Lately, mutations potentially associated with CDV resistance among the MPXVs of the 2022 outbreak have been reported, too.^14^ The prevalence of mutated MPXV may further rise once BCV was more widely and frequently used, which is also true for other orthopoxviruses because the mechanism of CDV resistance seems to be shared among the genus.^30^ Given the pacing threats posed by MPXV, variola and other emerging orthopoxviruses to human health,^31–34^ it is urgent to develop safer and more effective drugs.

CDV [(S)-1-(3-hydroxy-2-phosphonylmethoxypropyl)cytosine, (S)-HPMPC] and its prodrug BCV exemplify that the (S)-HPMP nucleoside can be a promising class of broad spectrum antiviral compounds.^9^ In fact, many other candidate molecules in this class have been synthesized and tested for antiviral effects. (S)-HPMPA [(S)-9-(3-hydroxy-2-phosphonomethoxypropyl)adenine] is one of these candidates, which showed broad spectrum antiviral activities in earlier studies.^9,35^ However, because of the poor oral bioavailability, (S)-HPMPA is considered unsuitable for clinical use. Great efforts have been made to modify (S)-HPMPA in order to increase its oral bioavailability and antiviral activity. Among these efforts, esterification of (S)-HPMP derivatives with alkoxyalkyl groups such as hexadecyloxypropyl (HDP) or octadecyloxyethyl (ODE) and conjugation with amino acid such as tyrosine or homoserinamide represent two attractive strategies.^36–48^ Nevertheless, only few candidate compounds, such as ODE-(S)-HPMPA and HDP-(S)-HPMPA, displayed promising in vivo anti-orthopoxvirus effects.^39,42^ Building upon the past efforts, we developed a novel method, esterification with formic acid, to improve the pharmaceutical characteristics of BCV, ODE-(S)-HPMPA and HDP-(S)-HPMPA (WO2024259930, WO2024259934). In this study, we systematically compared the antiviral activities of the novel prodrugs (BCV formate, ODE-(S)-HPMPA formate and HDP-(S)-HPMPA formate) with their parent drugs.

## Results

### In vitro cytotoxicity and in vivo pharmacokinetics of candidate compounds

**Fig. 1:**
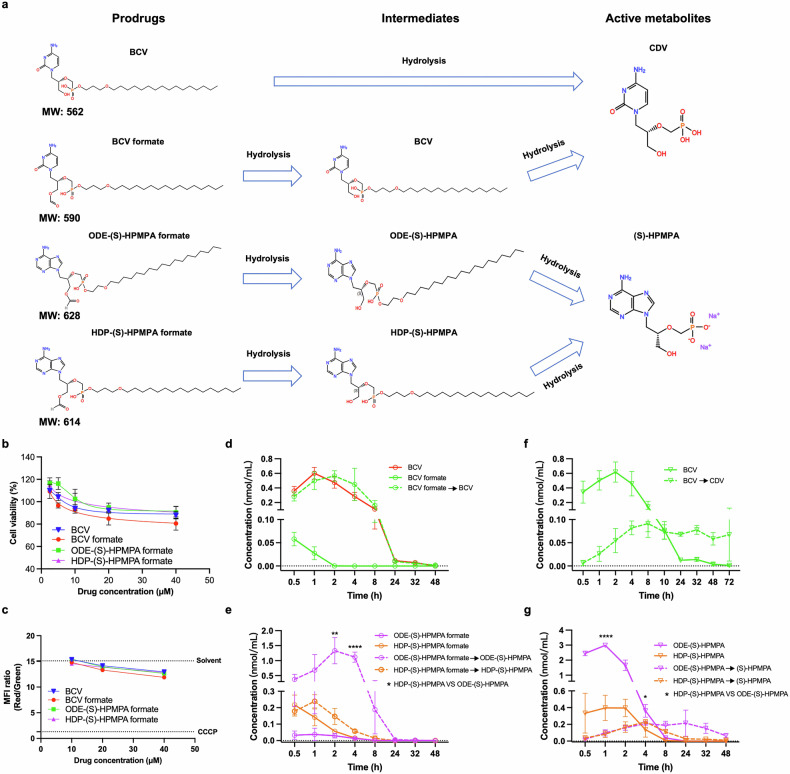
In vitro cytotoxicity and in vivo pharmacokinetic analyses of BCV and the candidate compounds. **a** The molecular structures and the theoretical metabolic pathways of BCV and the newly synthesized prodrugs. After two steps of hydrolysis, BCV formate can be converted to the same active parent drug (CDV) with BCV. ODE-(S)-HPMPA formate and HDP-(S)-HPMPA formate also take two steps of hydrolysis to turn into (S)-HPMPA. **b** Comparisons of cytotoxicities between candidate compounds and BCV. Limited by the solubility of BCV formate, ODE-(S)-HPMPA formate and HDP-(S)-HPMPA formate, the highest concentration employed in the cytotoxicity assay was 40 μM. Data are presented as mean ± SD. **c** Comparisons of mitochondrial toxicity between candidate compounds and BCV. The solvent and CCCP served as negative and positive control, respectively. **d**, **e** Plasma concentrations of BCV, BCV formate, ODE-(S)-HPMPA formate and HDP-(S)-HPMPA formate, as well as their respective one-step hydrolytic metabolites CDV, BCV, ODE-(S)-HPMPA and HDP-(S)-HPMPA, were measured at various time points following a single oral gavage dose (3 mice per group, the mice were administered 10 ml/kg of each prodrug at a concentration of 7.12 µmol/ml). **f**, **g** Plasma concentrations of BCV, ODE-(S)-HPMPA, and HDP-(S)-HPMPA, as well as their hydrolytic metabolites (CDV and (S)-HPMPA) were measured at various time points following a single oral gavage dose (3 mice per group, the mice were administered 10 ml/kg of each prodrug at a concentration of 7.12 µmol/ml). Data are presented as mean ± SD. Statistical significance is indicated as follows: *p < 0.05; **p < 0.01; ****p < 0.0001

The molecular structures of BCV and the three synthesized compounds, along with their active metabolites, are shown in Fig. 1a. BCV formate is a derivative of CDV, and ODE-(S)-HPMPA formate and HDP-(S)-HPMPA formate are prodrugs of (S)-HPMPA. Due to solubility limitations, the maximum concentration of the synthesized compounds (BCV formate, ODE-(S)-HPMPA formate, and HDP-(S)-HPMPA formate) used in vitro assays was restricted to 40 μM. All candidate compounds exhibited 50% cytotoxic concentration (CC_50_) values > 40 μM, as cell viabilities remained above 80% at this concentration (Fig. 1b). Mitochondrial toxicity was evaluated using a mitochondrial membrane potential assay kit with JC-1. At a concentration of 10 μM, none of the compounds affected mitochondrial membrane potential, while slight reductions in mitochondrial membrane potential were observed at higher concentrations (Fig. 1c).

To facilitate in vivo antiviral activity evaluation, we monitored the kinetics of absorption and metabolism for each compound after a single dose of oral gavage in mice. Our data showed that all compounds or their metabolites were detectable at a half hour post oral administration (Fig. 1d, e), suggesting that they could be quickly assimilated into blood. After absorption, BCV formate, ODE-(S)-HPMPA formate and HDP-(S)-HPMPA formate were instantly hydrolyzed into BCV, ODE-(S)-HPMPA and HDP-(S)-HPMPA, respectively. And the concentrations of these metabolites in blood peaked around 1 to 2 h post oral administration (Fig. 1d, e). Then, BCV was gradually hydrolyzed into CDV within 24 h post oral administration (Fig. 1f). While ODE-(S)-HPMPA and HDP-(S)-HPMPA were hydrolyzed faster into (S)-HPMPA, which lasted only 8 h (Fig. 1g).

### ODE-(S)-HPMPA formate and HDP-(S)-HPMPA formate show superior anti-orthopoxvirus activities than BCV and BCV formate

**Fig. 2:**
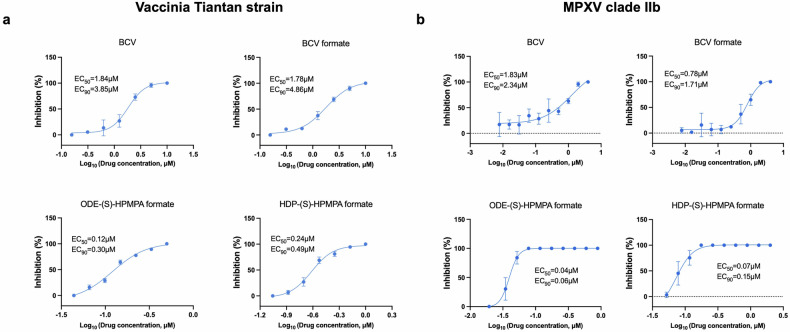
In vitro evaluations of anti-vaccinia and anti-MPXV activities of BCV and the candidate compounds. Vero cells were infected with vaccinia Tiantan strain (**a**) or MPXV (**b**) and treated with the indicated concentrations of BCV or the candidate prodrugs. All compounds were tested in triplicated wells at each concentration. The solvent was used as the non-treated control. The antiviral effects were determined by calculating the reduction in plaque formation. Data are presented as mean ± SD

To compare the anti-orthopoxvirus effects of the candidate drugs with BCV, we firstly tested their antiviral activities against vaccinia Tiantan strain and MPXV in vitro. Generally, the EC_50_ values of BCV observed in this study (Fig. 2a) were close to the previously reported range.^49^ The EC_50_ and EC_90_ of BCV formate against Tiantan vaccinia were similar with those of BCV, while the EC_50_ and EC_90_ of ODE-(S)-HPMPA formate and HDP-(S)-HPMPA formate were conspicuously lower (Fig. 2a). The anti-MPXV activity of BCV was similar with its effect against Tiantan vaccinia, while the other three compounds inhibited MPXV even more potently than Tiantan vaccinia (Fig. 2b). ODE-(S)-HPMPA formate demonstrated the most potent anti-MPXV effect, whose EC_50_ and EC_90_ were more than 40-fold lower than those of BCV (Fig. 2b). We also compared the in vitro anti-vaccinia virus activities of ODE-(S)-HPMPA formate and HDP-(S)-HPMPA formate to their non-formylated counterparts. The results demonstrated that the EC_50_ values of the formylated prodrugs decreased by 2- to 3-fold compared to their parent drugs, indicating that the esterification with formic acid improves the antiviral potencies of ODE-(S)-HPMPA and HDP-(S)-HPMPA (supplementary Fig. 1).

**Fig. 3:**
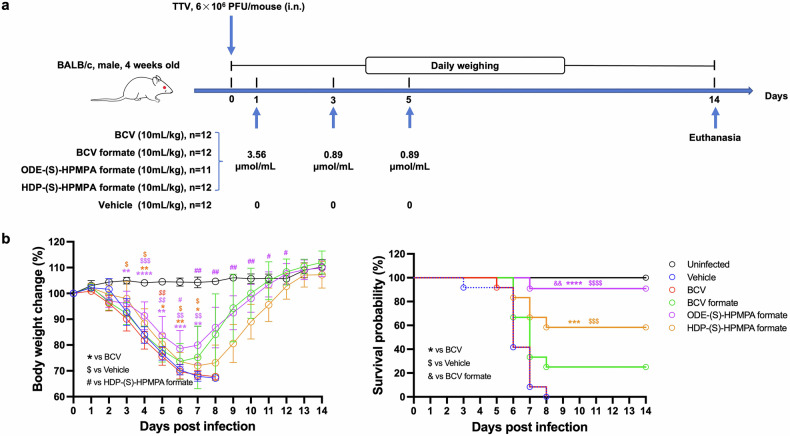
ODE-(S)-HPMPA formate and HDP-(S)-HPMPA formate showed superior therapeutic efficacies against lethal Tiantan vaccinia infection in mice. **a** The schematic illustration of experimental design. **b** Weight loss and survival rates of mice after being intranasally infected with the vaccinia Tiantan strain. Mice received the solvent served as the non-treated control. Weight loss ≥30% of initial body weight was recorded as ethical death and was therefore not represented in the figure. The data of weight loss are presented as mean ± SD. Statistical significance is indicated as follows: *, $, #: *p* < 0.05; **, $$, ##: *p* < 0.01; ***, $$$, ###: *p* < 0.001; ****, $$$$, ####: *p* < 0.0001

Next, we evaluated the in vivo anti-orthopoxvirus activities of the synthesized compounds using a lethal mouse model of vaccinia virus infection. As shown in Fig. 3a, 4 weeks old BALB/c mice were intranasally infected with 6 × 10^6^ PFU of vaccinia Tiantan strain. Equal moles of candidate drugs were given at 1 day, 3 days and 5 days post infection via oral gavage. All groups of infected mice experienced obvious body weight loss, which peaked around 7 to 8 days post infection for mice treated with BCV formate, ODE-(S)-HPMPA formate or HDP-(S)-HPMPA formate (Fig. 3b). In contrast, mice received the vehicle or BCV underwent more severe weight loss and all mice in these two groups ethically died by day 8 (Fig. 3b). Uninfected mice treated with each candidate compound showed no signs of weight loss (supplementary Fig. 2). For animals in the group treated with ODE-(S)-HPMPA formate, more than 90% of mice (10 out of 11) survived, a significant improvement in the survival rate compared with animals in all other groups (vehicle, BCV or BCV formate groups; Fig. 3b). The second-best drug was HDP-(S)-HPMPA formate, which protected significantly more mice from death than vehicle and BCV (Fig. 3b). BCV formate also displayed better protection, but its efficacy did not significantly differ from those of the vehicle and BCV (Fig. 3b).

**Fig. 4:**
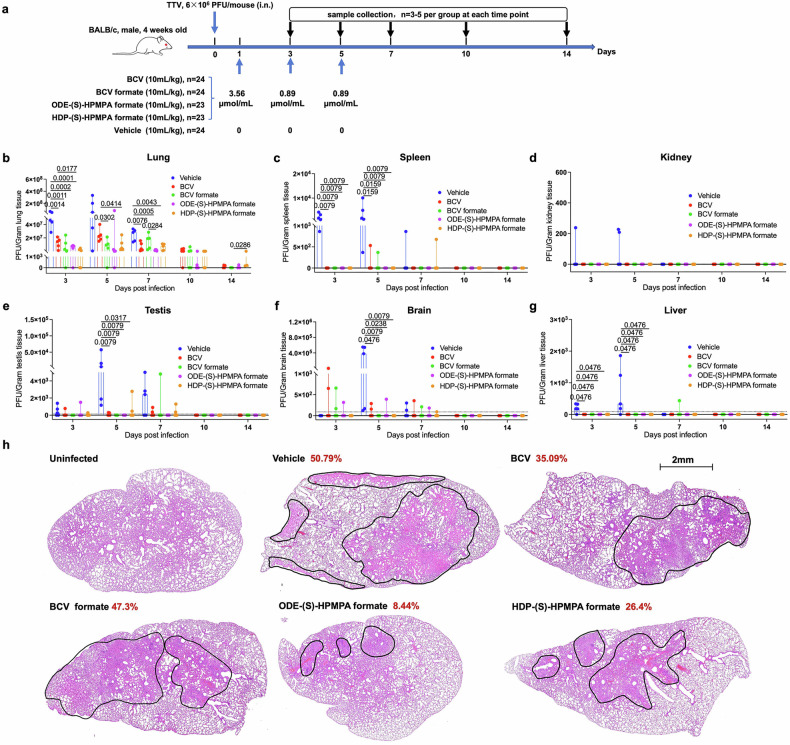
ODE-(S)-HPMPA formate and HDP-(S)-HPMPA formate more effectively reduced lung vaccinia virus titers and mitigated pathological severity compared to BCV and BCV formate. **a** The schematic illustration of experimental design. **b**–**g** Viral titers in the lung, spleen, kidney, brain, testis, and liver were measured by plaque assays at 3, 5, 7, 10, and 14 days post-infection, respectively. **h** Histopathological examination of lung tissue at 7 days post infection. Black outlines indicate areas of observed pathology, and the percentages of the lesion area relative to the total area of the tissue are denoted in red

To characterize the in vivo anti-vaccinia virus activities further, we repeated vaccinia virus infection and drug treatment in mice, but this time 3–5 mice of each group were euthanized at indicated time points for detections of viral replication, host responses and drug metabolites (Fig. 4a). We found that vaccinia virus resided mainly in lungs after intranasal infection and significant suppression of the virus by candidate drugs could be observed in all detected tissues except kidney (Fig. 4b–g), where live vaccinia viruses were rarely detected even in the non-treated group. Compared to BCV, BCV formate, ODE-(S)-HPMPA formate and HDP-(S)-HPMPA formate tended to decrease vaccinia virus titers in lungs, but statistical significance was only observed between BCV and ODE-(S)-HPMPA formate at day 3 and between BCV and HDP-(S)-HPMPA formate at day 5 (Fig. 4b). Histopathological examination showed that ODE-(S)-HPMPA formate and HDP-(S)-HPMPA formate diminished the size of lung lesions caused by vaccinia infection compared to BCV and BCV formate at 7 days post infection (Fig. 4h). Meanwhile, no signs of infection-caused or drug-related pathology were observed in other organs, including the kidney, spleen, testis, liver and brain (supplementary Fig. 3). We also monitored the cytokine responses in the lungs at 3, 5, 7, 10 and 14 days post infection. We found that different drugs impacted lung inflammation differentially (supplementary Fig. 4). Of note, at 7 days post vaccinia infection (approximately the nadir of mouse body weight), the transcription levels of IL-6—a key pro-inflammatory mediator in acute organ injury and sepsis, as well as an early biomarker of lung injury^50^—were significantly lower in the lungs of mice treated with ODE-(S)-HPMPA formate than those of mice treated with vehicle or BCV (supplementary Fig. 4a). While, the levels of IL-12, which promotes protective antiviral immunity,^51^ and TNF-α, crucial for regulating pulmonary inflammation during orthopoxvirus infections,^52^ were significantly higher in mice treated with ODE-(S)-HPMPA formate or BCV formate compared to the vehicle, HDP-(S)-HPMPA formate or BCV groups (supplementary Fig. 4b, c). These findings were partially in accordance with a previous study suggesting that (S)-HPMPA could activate TNF-α secretion.^53^

Pharmacokinetic analysis demonstrated that the metabolite of HDP-(S)-HPMPA formate was undetectable in the lung and spleen of mice at 2 or more days post-treatment (supplementary Fig. 5), which might be partly because it can be almost completely cleared from peripheral circulation within 24 h (Fig. 1g). The better in vivo efficacy of HDP-(S)-HPMPA formate compared to BCV and BCV formate is likely attributed to its more effective inhibition of viral infection (Fig. 2). We noticed that the liver contained the highest concentrations of drug metabolites during the whole process of drug treatments (supplementary Fig. 5). Hence, we assessed the liver toxicities of candidate drugs via measuring the serum ALT levels at day 7 (2 days post the third dose of drug treatment). The results showed that the average levels of serum ALT ranked as follows: HDP-(S)-HPMPA formate > BCV formate > BCV > vehicle > ODE-(S)-HPMPA formate > uninfected control (supplementary Fig. 6). Of note, the ALT levels of BCV, BCV formate and HDP-(S)-HPMPA formate treated mice were significantly higher than that in uninfected controls, while no significant difference was observed among mice of ODE-(S)-HPMPA formate treated group, vehicle group, and control group (supplementary Fig. 6).

### ODE-(S)-HPMPA formate and HDP-(S)-HPMPA formate can suppress HSV-1 both in vivo and in vitro, but are less effective than BCV and BCV formate in protecting mice against lethal infection

**Fig. 5:**
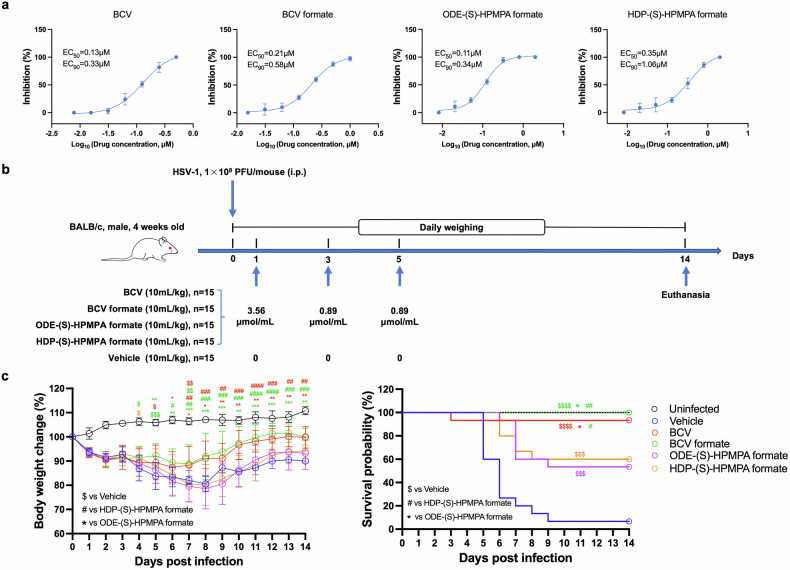
Evaluations of in vivo and in vitro anti-HSV-1 potencies of BCV and the candidate prodrugs. **a** Vero cells were infected with HSV-1 stain 17 and treated with indicated concentrations of BCV or the candidate compounds. All compounds were tested in triplicated wells at each concentration. The solvent was used as the non-treated control. The antiviral effects were determined by calculating the reduction in plaque formation. Data are presented as mean ± SD. **b** The schematic illustration of in vivo experiment design. **c** Weight loss and survival rates of mice after being intraperitoneally infected with HSV-1. Mice received the solvent served as the non-treated control. The data of weight loss are presented as mean ± SD. Statistical significance is indicated as follows: *, $, #: *p* < 0.05; **, $$, ##: *p* < 0.01; ***, $$$, ###: *p* < 0.001; ****, $$$$, ####: *p* < 0.0001

As NAs usually have broad spectrum antiviral activity,^54^ we next compared the anti-HSV-1 activities between the candidate drugs and BCV. The results of in vitro antiviral assays indicated that the EC_50_ and EC_90_ of BCV formate and ODE-(S)-HPMPA formate against HSV-1 were close to those of BCV, while the EC_50_ and EC_90_ of HDP-(S)-HPMPA formate were slightly higher (Fig. 5a). To validate this observation, we tested their efficacies against HSV-1 lethal infection in vivo. As shown in Fig. 5b, BALB/c mice were intraperitoneally infected with 1 × 10^8^ PFU of HSV-1 (strain-17) followed by the same regimen of drug treatment with vaccinia infected mice. Our data showed that BCV and BCV formate alleviated mouse body weight loss, whereas ODE-(S)-HPMPA formate and HDP-(S)-HPMPA formate apparently did not (Fig. 5c). Weight loss data for each individual mouse were shown in supplementary Fig. 7 to better illustrate the variation in weight loss within the HSV-1 infection group. Albeit ODE-(S)-HPMPA formate and HDP-(S)-HPMPA formate provided significant protection against lethal HSV-1 infection than the vehicle, their protection rates were significantly lower than those of BCV and BCV formate (Fig. 5c). It is intriguing to see that the adenine analogs (ODE-(S)-HPMPA formate and HDP-(S)-HPMPA formate) were more effective in protecting mice against vaccinia infection while less effective towards HSV-1 infection compared to cytidine analogs (BCV and BCV formate). We further compared the genomic DNA base composition of orthopoxvirus with that of HSV and found that orthopoxviruses contain obviously less GC than HSV (supplementary Fig. 8 and supplementary Table 1). This difference might lead to that adenine analogs would have more chance to be incorporated into orthopoxvirus genome, while the cytidine analogs would be more frequently incorporated into HSV genome.

Inspired by this observation, we were curious whether a combined regimen of purine and pyrimidine analogs would provide more balanced protection against orthopoxvirus and herpesvirus. To test this hypothesis, we treated vaccinia infected mice with the equal molar mixture of BCV formate and ODE-(S)-HPMPA formate, and treated HSV-1 infected mice with the equal molar mixture of BCV formate and ODE-(S)-HPMPA formate (supplementary Fig. 9a). The total dosage for each mixed regimen was maintained in consistency with the aforementioned single-drug regimen. The result showed that all mice survived after being treated with the combined regimens (supplementary Fig. 9b), which was obviously better than any single-drug treatment (Figs. 3 and 5).

### Three candidate compounds exhibit better in vitro anti-hAdV activity than BCV

**Fig. 6:**
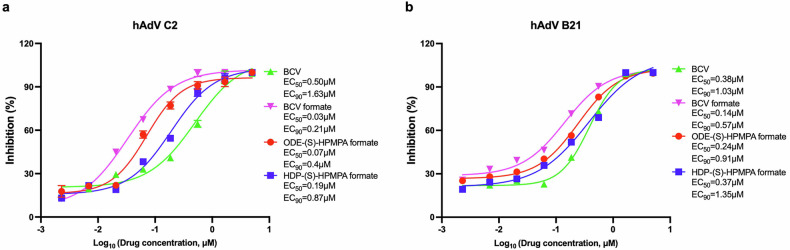
In vitro evaluation of anti-hAdV activities of BCV and the candidate compounds. Vero cells were infected with either hAdV C2 (**a**) or hAdV B21 (**b**) and subsequently treated with indicated concentrations of BCV or the candidate prodrugs. All compounds were tested in quadruplicate at each concentration. The solvent was used as the non-treated control. Data are presented as mean ± SD

BCV has been used compassionately to treat adenovirus infection for pediatric patients receiving hematopoietic stem cell transplantation.^55^ In this study, we compared the in vitro anti-human adenovirus (hAdV) potencies between three candidate prodrugs and BCV using clinically isolated strains of hAdV type C2 and type B21. The EC_50_ values of the three candidates, especially those of BCV formate and ODE-(S)-HPMPA formate, were much lower than the EC_50_ values of BCV (Fig. 6). Considering that the liver toxicity of ODE-(S)-HPMPA formate were significantly lower than BCV formate (supplementary Fig. 6), we thought ODE-(S)-HPMPA formate could be a better candidate therapeutic for hAdV infections.

## Discussion

NAs have been widely used to treat cancer and viral diseases.^56^ Given that the catalytic units of viral polymerases are conserved in their structures,^57^ NAs are thought to be an important class of broad-spectrum antiviral agents.^54^ Since the first antiviral NA was approved in 1969, more than two dozen NAs have been licensed to treat viral infections.^56^ However, only a limited number of viral diseases are targeted.^7^ Among the six families of double stranded DNA viruses (Hepadnaviridae, Polyomaviridae, Papillomaviridae, Adenoviridae, Herpesviridae and Poxviridae) known to cause human diseases,^58^ only five viruses from three families (Hepadnaviridae, Herpesviridae and Poxviridae) can be treated with approved NAs.^7,8^ BCV is currently the only NA that can be used to treat human Mpox disease under an FDA-authorized single-patient e-IND, while its efficacy is still inconclusive.^59^ In this work, we and collaborators synthesized a new precursor of CDV and two novel precursors of (S)-HPMPA and evaluated their inhibitory activities against multiple double-stranded DNA viruses, including orthopoxviruses, HSV-1 (strain 17) and human adenoviruses. Through the experiments shown here, we found that BCV and three candidate prodrugs exhibited varied antiviral potencies against different DNA viruses. The anti-orthopoxvirus potency of BCV formate was non-inferior to BCV, by contrast the two prodrugs of (S)-HPMPA were remarkably more effective than BCV in suppressing orthopoxviruses both in vitro and in vivo. ODE-(S)-HPMPA formate was the best-performing anti-orthopoxvirus compound, which displayed the most robust anti-orthopoxvirus activity and the lowest hepatotoxicity.

As ODE-(S)-HPMPA formate and HDP-(S)-HPMPA formate were designed based on previously reported ODE-(S)-HPMPA and HDP-(S)-HPMPA,^39,42^ we further compared their antiviral activities with the parent drugs and confirmed that esterification with formic acid conspicuously improved the in vitro antiviral activity of ODE-(S)-HPMPA and HDP-(S)-HPMPA (supplementary Fig. 1). We did not compare their in vivo antiviral effects with the parent drugs, but compared to the data shown in the literature,^42^ both ODE-(S)-HPMPA formate and HDP-(S)-HPMPA formate are much more efficacious than their counterparts in protecting mice from lethal vaccinia infection (supplementary Table 2). The fact that ODE-(S)-HPMPA formate was more efficacious than HDP-(S)-HPMPA formate in protecting mice against vaccinia infection might be because the active metabolite of ODE-(S)-HPMPA formate distributed to lung more efficiently and steadily than that of HDP-(S)-HPMPA formate (supplementary Fig. 5). We did not investigate the viral mutations induced by three novel prodrugs, because previous studies have comprehensively characterized the mutations in viral DNA polymerase (e.g., A314V/T, A684V, and S851Y) that confers resistance against (S)-HPMPA (the active metabolite of ODE-(S)-HPMPA formate and HDP-(S)-HPMPA formate) and (S)-HPMPC (the active metabolite of BCV formate).^60,61^ We think that the resistant mutation patterns induced by the three novel prodrugs should be similar with those induced by their parent drugs. Moreover, since ODE-(S)-HPMPA formate and HDP-(S)-HPMPA formate exhibited enhanced efficacies against orthopoxviruses compared to their parent compounds, we speculate that these novel prodrugs may further reduce the likelihood of resistance development.

A second key finding of this work is that BCV and BCV formate provided significantly stronger protection against lethal HSV-1 infection than ODE-(S)-HPMPA formate and HDP-(S)-HPMPA formate in mice. We did not test the antiviral activity of the three new prodrugs against HSV-2, firstly because the infection prevalence of HSV-1 is much higher than that of HSV-2 globally,^62^ and secondly because the DNA polymerase of HSV-1 shares high amino acid homology with that of HSV-2.^63^ Therefore, we extrapolate that the activity of the new prodrugs against HSV-1 and HSV-2 might be similar. The primary antiviral mechanism of NAs is that they resemble natural nucleos(t)ides and act by being incorporated into the nascent DNA chain,^64,65^ however the mechanism underlying the varied protective effects against orthopoxvirus and HSV-1 is not clear. As herpesvirus genome contains obviously more GC than orthopoxvirus genome, we speculate that the cytidine analogs (BCV and BCV formate) might be more effective in suppressing herpesviruses, while adenine analogs, ODE-(S)-HPMPA formate and HDP-(S)-HPMPA formate, might inhibit orthopoxviruses more efficiently. Consistent with this hypothesis, we proved that combined regimens of cytidine and adenine analogs could provide excellent protection against both vaccinia and HSV-1 infection in mice, indicating that combination of purine and pyrimidine analogs might be a rational and promising strategy to develop a broad-spectrum antiviral therapy. Inconsistent with in vivo results, in vitro antiviral assays showed similar inhibitory effects of BCV and ODE-(S)-HPMPA formate on HSV-1, which might be due to that the relatively low dose of HSV-1 used in vitro is not adequate to discriminate the anti-HSV-1 potencies of these two prodrugs. While, in the lethally infected mice, the difference between their protective effects can be more clearly observed.

Finally, we tested the anti-hAdV potencies of the candidate prodrugs through in vitro experiments. Adenoviruses can cause life-threatening disease not only in immunocompromised patients but also in the healthy, although the latter occurs less frequently.^66,67^ Both CDV and BCV have been compassionately used to treat hAdV infections in children receiving hematopoietic cell transplantation,^55,68^ but there are no officially approved therapeutics. Here we showed ODE-(S)-HPMPA formate could be a good anti-hAdV agent, which exhibited a good safety profile and relatively stronger inhibitory effect than BCV.

A major limitation of this work is that we did not test the in vivo anti-MPXV and anti-hAdV effects of the candidate prodrugs, primarily because no appropriate animal models are available to us. Evaluations using an MPXV infected rhesus macaque model^69^ and an hAdV infected human lung organoid model^70^ may provide more relevant data before moving any of the candidate prodrugs into clinical trials. Besides, as we did not include any drugs approved for the treatment of herpes in this study, we cannot tell whether the novel prodrugs, especially BCV formate, would be a competitive alternative for herpes treatment.

## Materials and methods

### Ethical statement

Experiments using mice were approved by the Research Ethics Review Committee of the Shanghai Public Health Clinical Center Affiliated to Fudan University. Experiments using live vaccinia, HSV-1 and adenoviruses were conducted in a BSL-2 or ABSL-2 lab. Measurements of in vitro inhibition of MPXV were performed in a BSL-3 lab.

### Antiviral compounds

BCV (CAS number 444805-28-1, purity 99.82%) was purchased from Shanghai Haohong Scientific Co., Ltd.. Compound BCV formate (purity 97.86%), ODE-(S)-HPMPA formate (purity 95.03%) and compound HDP-(S)-HPMPA formate (purity 96.97%) were synthesized by our collaborator, Risen (Shanghai) Pharma Tech Co., Ltd. The synthesis protocols for these three prodrugs, along with their HPLC purity spectra, ¹H NMR and mass spectra, are provided in Supplementary Materials.

### Virus propagation and titration

Vaccinia Tiantan strain was propagated in Vero cells. Briefly, confluent Vero cells plated in 100 × 10 mm petri dishes were inoculated with Tiantan vaccinia at a multiplicity of infection (MOI) of 0.01. After 2 h absorption at 37 °C with 5% CO_2_, the inoculum was removed and 10 ml of fresh maintenance medium (DMEM containing 3% FBS and 1% PS) was added to each dish. The cell cultures were maintained at 37 °C with 5% CO_2_ for another 2 days. After that, propagated vaccinia viruses were harvested by three rapid freeze-thaw cycles of the infected cells. HSV-1 propagation followed a similar protocol except that the inoculum was adsorbed for 1 h with gentle agitation at room temperature and the propagated virus was harvested from the culture supernatant by centrifuging at 34,000 *g* for 2 h at 4 °C.^71^ MPXV was sourced from National Kunming High-level Biosafety Primate Research Center, Institute of Medical Biology, Chinese Academy of Medical Sciences and Peking Union Medical College. Viruses were amplified in Vero cells, cultured in DMEM supplemented with 2% FBS and 1% PS.

Harvested vaccinia, MPXV and HSV-1 viruses were titrated using plaque assays. Briefly, confluent monolayers of Vero cells in 24-well plates were infected with 10-fold serial dilutions of each virus, ranging from 10^−1^ to 10^−9^. For vaccinia viruses and MPXV, the plates were incubated at 37 °C with 5% CO_2_ for 2 h. For HSV-1 (strain-17), the plates were gently agitated at room temperature for 1 h. After absorption, the cells were overlaid with 800 μL of overlay medium (DMEM containing 1% FBS and 1.25% methyl cellulose) and incubated at 37 °C with 5% CO_2_ for 4 days. Post-incubation, the plates were fixed with 4% neutral formalin and stained with crystal violet. Plaques were visually counted, and the viral titers were calculated and expressed as plaque-forming units (PFU) per milliliter.

HAdV-B21 and C2 were isolated from nasopharyngeal swab specimens of HAdV-positive patients and propagated in HEp-2 cells. Briefly, HEp-2 cells were plated in 100 × 10 mm petri dishes and inoculated with hAdVs. After a 1-hour absorption at 37 °C with 5% CO_2_, the inoculum was removed, and 10 mL of maintenance medium was added to each dish. The cultures were maintained at 37 °C with 5% CO_2_ until more than 80% of the cells exhibited cytopathic effects (CPE). Propagated hAdVs were harvested by three rapid freeze-thaw cycles of the infected cells. The stocks of hAdVs were titrated using the TCID_50_ assay. Briefly, confluent monolayers of Vero cells in 96-well cell culture plates were infected with 10-fold serial dilutions of the hAdVs. The wells were monitored for the presence of CPE and the infectious adenovirus titer was determined as TCID_50_ per mL using the Spearman-Karber method.

### Cytotoxicity assay

The cytotoxicities of candidate compounds were determined using a Promega CellTiter-Glo® Luminescent Cell Viability Assay kit (Cat# G7571, Madison, WI, USA) according to the manufacturer’s instructions. Briefly, Vero cells were seeded in 96-well plates and incubated overnight. The candidate compounds were firstly dissolved in a mixed solvent (Dichloromethane: Methanol, v: v = 1:3) and then serially diluted in maintenance medium before adding to the wells. The solvent was used as the non-treated control. The final solvent concentration was kept consistent across all wells at 2%. Each concentration was tested in quadruplicate. The plates were incubated for 96 h at 37 °C with 5% CO_2_. Subsequently, reconstituted Steady-Glo reagent was added and assay plates were incubated for 10 min at room temperature. The luminescence was measured using a luminescence microplate reader (GloMax® Navigator Microplate Luminometer, Promega, USA). Wells containing culture medium without cells served as blank controls. Cell viabilities were calculated using the following equation: Cell viability (%) = (drug treated well - blank) / (solvent well - blank) × 100%.

### Mitochondrial toxicity assay

Mitochondrial membrane potential was determined using Mitochondrial membrane potential assay kit with JC-1 (Cat# C2006, Beyotime Institute of Biotechnology, China) according to the manufacturer’s instructions. Briefly, Vero cells were seeded into 6-well plates and incubated overnight. Candidate compounds were prepared as described in the Cytotoxicity Assays section and added to the wells. The plates were incubated for 4 days at 37 °C with 5% CO_2_. After incubation, the cells were harvested and resuspended in JC-1 staining buffer, followed by incubation for 20 min at 37 °C with 5% CO_2_. Post-staining, the cells were washed and analyzed using a BD Fortessa flow cytometer. The data were processed and analyzed with FlowJo software version 10 (TreeStar Inc., Ashland, OR, USA). Mitochondrial membrane potential was assessed by calculating the ratio of mean fluorescence intensity (MFI) of red to green fluorescence. The solvent was used as the non-treated control and cells treated with 10 µM Carbonyl Cyanide m-chlorophenylhydrazone (CCCP) for 10 min served as the positive control.

### In vitro viral inhibition assays

Twenty PFU of orthopoxvirus (Tiantan vaccinia or MPXV) or HSV-1 virus suspended in 200 μL maintenance medium were transferred onto confluent Vero cell monolayers in 24-well plates. For vaccinia virus, the plates were incubated at 37 °C with 5% CO_2_ for 2 h. For HSV-1, the plates were gently agitated at room temperature for 1 h. 800 μL of overlay-medium containing DCM:MeOH (v:v = 1:3) or serially diluted candidate compounds was added to each well after removing the inoculums. The plates were then incubated for 4 days at 37 °C with 5% CO_2_. Finally, the plates were fixed with 4% neutral formalin and stained with crystal violet. The number of plaques in each well was visually counted. All compounds were tested in quadruplicates at each dilution. The viral inhibition rate was calculated using the formula: Inhibition rate (%) = (1 − the average plaque number of the sample wells / the average plaque number of the solvent wells) × 100%.

In vitro inhibition of hAdV B21 and C2 were performed as described previously.^72^ Briefly, 25,000 Vero cells were plated in each well of a 96-well cell culture plate, followed by addition of diluted adenovirus (with 50 and 200 TCID_50_/well for B21 and C2, respectively) and serially diluted candidate compounds. The solvent was used as the non-treated control. The plates were then incubated at 37 °C with 5% CO_2_ for 4 days. Cytopathology was quantitively assessed using a Promega CellTiter-Glo® Luminescent Cell Viability Assay kit. The viral inhibition rate was calculated using the formula: Inhibition rate (%) = (drug treated well - blank) - (non-treated control well - blank) × 100%.

### In vivo evaluation of antiviral effects

We established mouse infection models to evaluate the antiviral effects of the candidate compounds against Tiantan vaccinia and HSV-1, respectively. Vaccinia Tiantan strain (6 × 10^6^ PFU) was suspended in 40 μL 1×PBS and instilled intranasally into 4-week-old male BALB/c mice under transient anesthesia induced by inhaled isoflurane (Cat# R510-22, RWD Life Science, China). For HSV-1 infections, 4-week-old female BALB/c mice were infected intraperitoneally with 1 × 10^8^ PFU of HSV-1 suspended in 200 μL of 1×PBS. After infection, mice were weighed daily to monitor disease progression. In the survival observation presented in Figs. 3, 5 and supplementary Fig. 9, the ethical endpoint for the Tiantan vaccinia infected group was defined as ≥30% weight loss, while for the HSV-1 infected group, the endpoint is biological death, because all HSV-1 infected mice, including the succumbed ones, didn’t show ≥30% weight loss. However, in the experiment shown in Fig. 4, supplementary Figs. 4 and 5, in order to monitor the in vivo viral replication and pathological consequences dynamically, all the mice, no matter how severe the weight loss was, were maintained and sampled at predefined time points (days 3, 5, 7, 10 and 14) unless the mice died biologically.

For treatment experiments, mice were given equal moles of candidate compounds dissolved in saline containing 5% DMSO, 10% Solutol, and 17% SBE-β-CD via oral gavage at days 1, 3 and 5 post-infection as specified in Figs. 3–5. On day 1, the mice were administered 10 ml/kg of each prodrug at a concentration of 3.56 µmol/ml, followed by 10 ml/kg of each prodrug at a concentration of 0.89 µmol/ml on days 3 and 5. Control animals received an equivalent volume of the vehicle via the same gavage method. Infected mice were euthanized at timepoints indicated in Figs. 3–5.

### Pharmaceutical analyses of mouse tissue samples

Anticoagulated mouse blood samples were collected and centrifuged at 3200 rpm for 10 min at 4 °C to separate the plasma, which was then aliquoted and stored at −80 °C until analysis. Tissue samples, including spleen, brain, liver, kidney, lung, and testes, were homogenized and centrifuged at 4100 *g* for 15 min to obtain supernatants for pharmacokinetic analysis. The concentrations of the candidate compounds, intermediate metabolites, and active metabolites in both plasma and tissue samples were determined using a validated liquid chromatography-tandem mass spectrometry (LC-MS/MS) method, as described in the literature.^73^ The lower limits of quantitation (LLOQ) were 1 ng/mL in plasma, and in tissues, the LLOQs were 10 ng/g for ODE-(S)-HPMPA and BCV, 20 ng/g for CDV, and 50 ng/g for (S)-HPMPA and HDP-(S)-HPMPA.

### Titration of Tiantan vaccinia in mouse organs

Tissue samples were pre-cooled on ice and homogenized using high-throughput tissue grinding machine (Cat# Scientz-192, NingBo Scientz Biotechnology Co., Ningbo, China). After that, the homogenates were centrifuged at 2000 *g* for 10 min and the supernatants were collected for viral titration following similar procedures to those described above for in vitro viral titration. Briefly, confluent monolayers of Vero cells in 24-well plates were inoculated with 10-fold serially diluted supernatants of tissue homogenates. After incubation, the wells were overlaid with 800 μL of overlay medium and incubated for 4 days. Finally, the plates were fixed with neutral formalin and stained with crystal violet, and plaques were counted to determine viral titers.

### Histological examination

The mouse that exhibited the median weight loss of each group at 7 days post-Tiantan vaccinia infection was selected for histopathological examination of multiple organs. Anatomically matched tissue samples were collected from each mouse to ensure consistency, including the left upper lung lobe, left liver lobe, posterior part of spleen and whole organs of left kidney, cerebrum, and left testis. Collected tissues were fixed in 4% neutral paraformaldehyde for 2 h, followed by standard processing and embedding in paraffin. Tissue sections were prepared along the plane representing the maximum cross-sectional aera for each paraffin-embedded block and stained with hematoxylin and eosin (H&E). The stained slides were analyzed using the KF-PRO-120 digital pathology slide scanner (KFBIO) to assess tissue morphology and the extent of pathological changes. Lesion boundaries were defined by examining inflammatory cell infiltration, disruption of alveolar and bronchial structures, as well as interstitial edema and hemorrhage. The images were analyzed and exported by K-VIEWER1.5.5.6 software.

### Detection of cytokine transcription in mouse lung

Total RNA was extracted from mouse lung tissues collected at multiple timepoints post Tiantan vaccinia infection using Trizol reagent (Cat# 15596026CN, Life Technologies, USA). 1 μg of total RNA from each sample was reversely transcribed using the HiScript II Q RT SuperMix for qPCR (+gDNA wiper) (Cat# R223-01, Vazyme, China). Real-time PCR was conducted with the TB Green® Premix Ex Taq™ II (Tli RNase H Plus) (Cat# RR820Q, TaKaRa, China) using an ABI 7500 real-time fluorescence quantitative PCR (qPCR) instrument (Thermo Fisher Technology Co., LTD., USA). Transcription levels of cytokine genes relative to the GAPDH gene were calculated using the 2^−ΔCt^ method, where ΔCt = Ct of the target gene - Ct of GAPDH. The primers of mouse cytokines and GAPDH genes used for qRT-PCR are listed in supplemental Table 3, which were synthesized according to a previous work.^74^

### Determination of Alanine Aminotransferase (ALT) in murine serum

Peripheral blood was collected from mice at 2 days post the third dose of drug treatment. Serum was separated after clotting at room temperature for 1 h by centrifuging at 1000 *g* for 10 min at 4 °C. Serum ALT activity was determined using a commercialized ELISA kit following the manufacturer’s protocol (Cat# JL12668, Jianglai Biotechnology, Shanghai, China). Optical density was measured at 450 nm using a microplate reader (Cat# 800TS, Biotek, USA).

### Statistical methods

Statistical analyses were conducted using GraphPad Prism 9 (GraphPad Software, USA). The normality of the data was checked before all downstream statistical analyses. Comparisons between two groups were performed by the method of t-test. Differences among multiple groups were compared by the method of one-way ANOVA. EC_50_ and EC_90_ values were calculated using a four-parameter variable slope non-linear regression method. Survival curves were compared by the Cox-Mantel test. *P* ≤ 0.05 was considered as statistically significant.

## Supplementary information

Supplementary Materials

## Data Availability

The datasets generated and/or analyzed during the current study are available from the corresponding author upon reasonable request.
