# Correction: Novel derivatives of brincidofovir and (S)-9-(3-hydroxy-2-phosphonylmethoxypropyl)adenine inhibit orthopoxviruses and human adenoviruses more potently than brincidofovir

**DOI:** 10.1038/s41392-026-02809-y

**Published:** 2026-06-26

**Authors:** Yifan Zhang, Yanmin Wan, Cuiyuan Guo, Zhaoqin Zhu, Chao Qiu, Jiasheng Lu, Yanan Zhou, Jiaojiao Zheng, Fahui Dai, Xiaoyang Cheng, Kunlu Deng, Wanhai Wang, Youchun Wang, Wenhong Zhang

**Affiliations:** 1 Department of Infectious Diseases, Shanghai Key Laboratory of Infectious Diseases and Biosafety Emergency Response, National Medical Center for Infectious Diseases, Huashan Hospital, State Key Laboratory of Genetic Engineering, School of Life Science, Fudan University; Shanghai Sci-Tech Inno Center for Infection & Immunity, Shanghai, China; 2 Department of laboratory medicine, Shanghai Public Health Clinical Center, Shanghai, China; 3 School of Life Sciences, Fudan University, Shanghai, China; 4 Department of radiology, Shanghai Public Health Clinical Center, Shanghai, China; 5 Clinical Laboratory, The First Affiliated Hospital of Zhengzhou University, Key Laboratory of Laboratory Medicine of Henan Province, Zhengzhou, China; 6 Biosafety Level 3 Laboratory, Shanghai Public Health Clinical Center, Shanghai, China; 7 Institutes of biomedical sciences & Shanghai Key Laboratory of Medical Epigenetics, Fudan University, Shanghai, China; 8 Shenzhen Institutes of Advanced Technology, Chinese Academy of Sciences, Shenzhen, China; 9 Risen (Shanghai) Pharma Tech Co. Ltd., Shanghai, China; 10 Institute of Medical Biology, Chinese Academy of Medical Sciences and Peking Union Medical College, Kunming, China; 11 Key Laboratory of Pathogen Infection Prevention and Control (Peking Union Medical College), Ministry of Education; State Key Laboratory of Respiratory Health and Multimorbidity, Beijing, China

**Keywords:** Drug discovery, Drug development

Correction to: *Signal Transduction and Targeted Therapy* 10.1038/s41392-025-02207-w, published online 11 April 2025

After online publication of the article,^1^ the authors noticed inadvertent errors in the Materials and Methods section. In the “In vitro viral inhibition assays” subsection, the sentence “The solvent was used as the non-treated control.” has been corrected to “Infected cells treated with solvent only were used as the virus control, and uninfected cells treated with solvent only were used as the non-treated control.” The formula “Inhibition rate (%) = (drug treated well - blank) - (non-treated control well - blank) × 100%” has been corrected to “Inhibition rate (%) = [1 - (non-treated control well - drug-treated well)/(non-treated control well - virus control well)] × 100%.” In the “Histological examination” subsection, the misspelled word “aera” has been corrected to “area”.

In addition, the layout of the P value symbols in Figure 3b, Figure 4b, Figure 4c, Figure 4g, and Figure 5c was adjusted for alignment and clarity only, no numerical values, statistical analyses, or figure contents were changed.

**Figure 3 Incorrect** 
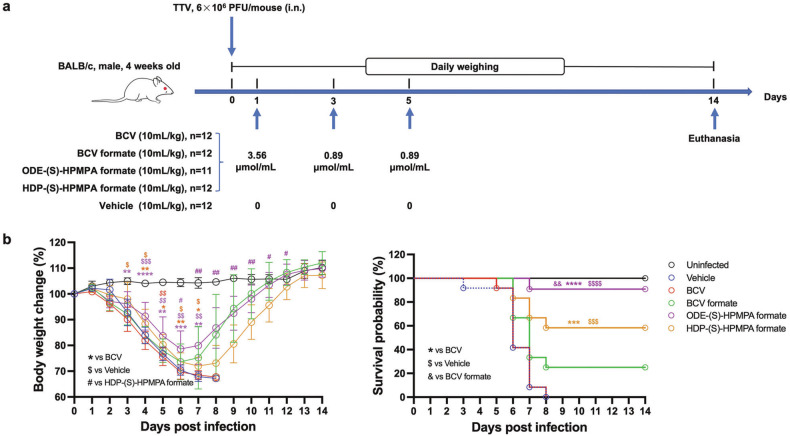


**Figure 3 Corrected** 
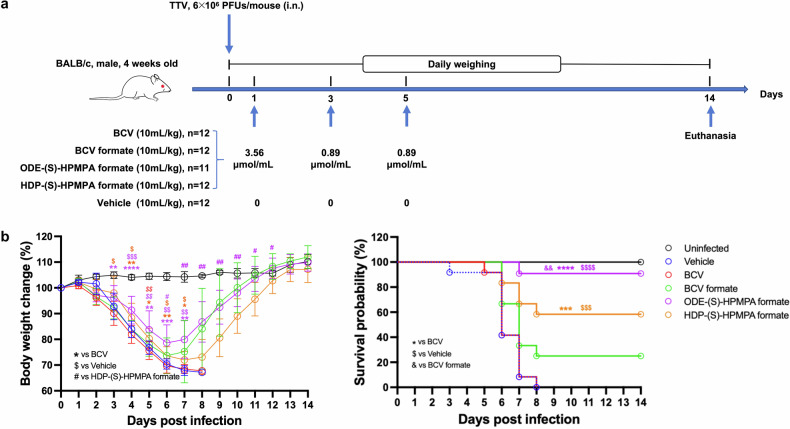


**Figure 4 Incorrect** 
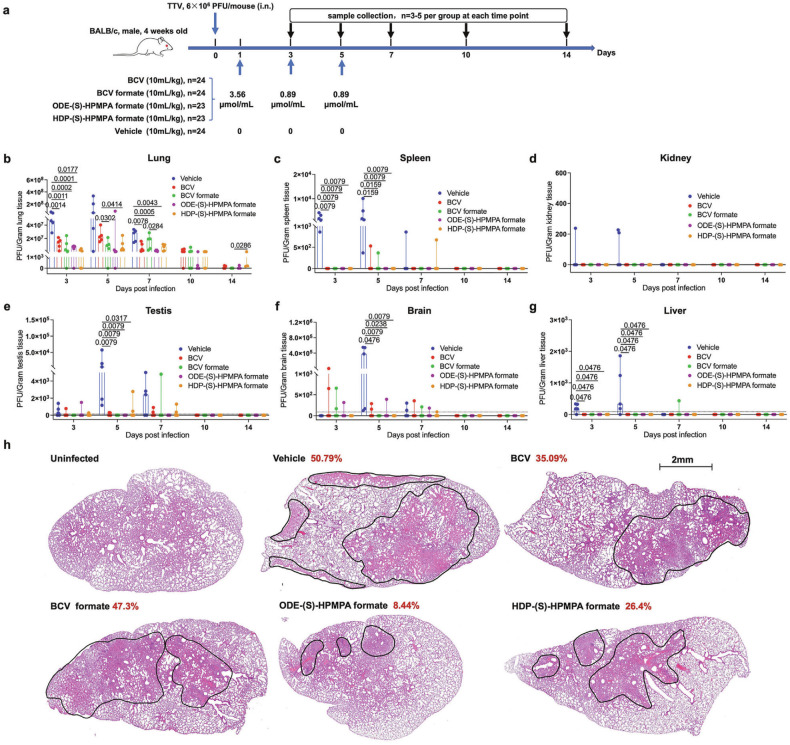


**Figure 4 Corrected** 
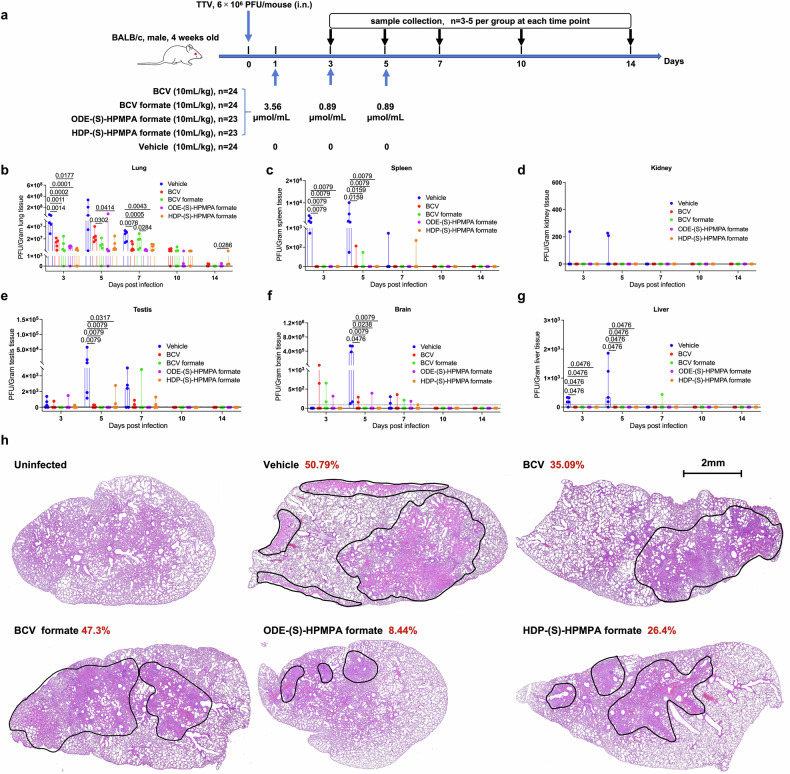


**Figure 5 Incorrect** 
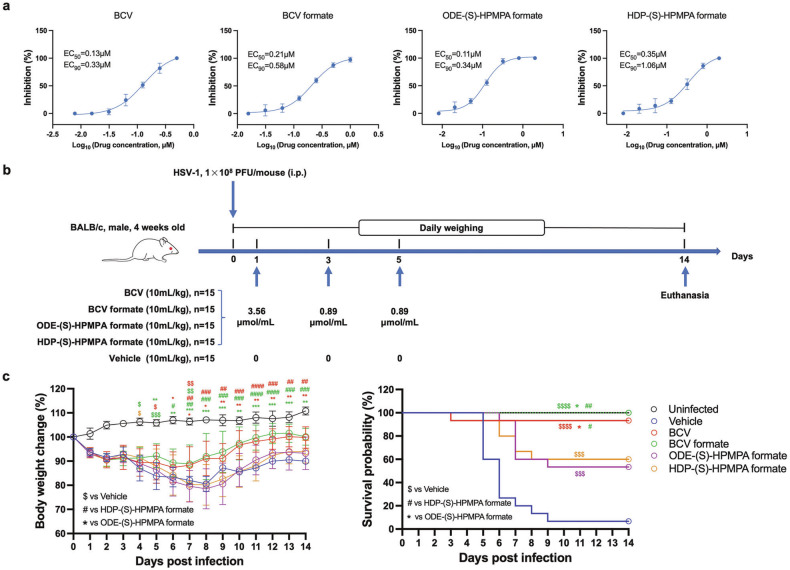


**Figure 5 Corrected** 
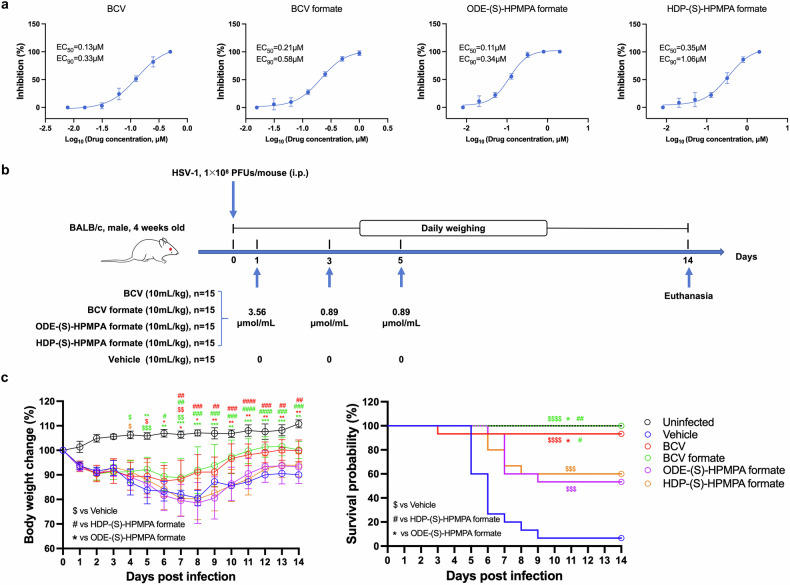


These corrections do not affect the results, interpretations, or conclusions of the study.
