# Supplementary material for: Novel derivatives of brincidofovir and (S)-9-(3-hydroxy-2-phosphonylmethoxypropyl)adenine inhibit orthopoxviruses and human adenoviruses more potently than brincidofovir
Source: Signal Transduct Target Ther. 2025 Apr 11;10:114. doi: 10.1038/s41392-025-02207-w (PMC11985979; doi:10.1038/s41392-025-02207-w)
Supplement: Supplementary file 1 — Supplementary Materials [file 41392_2025_2207_MOESM1_ESM.docx]

Supplementary Materials for

Novel derivatives of brincidofovir and (S)-9-(3-hydroxy-2-phosphonylmethoxypropyl)adenine inhibit orthopoxviruses and human adenoviruses more potently than brincidofovir

Yifan Zhang^1,2,7,#^, Yanmin Wan^1,3,*,#^, Cuiyuan Guo^2,4,#^, Zhaoqin Zhu^2,5,#^, Chao Qiu^6,#^, Jiasheng Lu^7,8,9^, Yanan Zhou^10^, Jiaojiao Zheng^5^, Fahui Dai^5^, Xiaoyang Cheng^1,2^, Kunlu Deng^1,2^, Wanhai Wang^4^, Youchun Wang^10,11,*^, Wenhong Zhang^1,*^

Correspondence to: yanmin_wan@fudan.edu.cn, [wangyc@nifdc.org.cn](mailto:wangyc@nifdc.org.cn), zhangwenhong@fudan.edu.cn

**This PDF file includes:**

Materials and Methods

Figures. S1 to S11

Tables S1 to S3

Materials and Methods

1. **The synthesis scheme and procedure of BCV formate**

A: To a mixture of NaH (4.91 g, 122.82 mmol, 60% purity, 1.5 eq.) in DMF (150 mL) was cooled to about 0 °C. The solution of propane-1,3-diol (28.04 g, 368.45 mmol, 26.63 mL, 4.5 eq.) in DMF (150 mL) was added into the mixture. The mixture was stirred at room temperature for 10 min, and then 1-bromohexadecane (25 g, 81.88 mmol, 25.03 mL, 1.0 eq.) was added into the mixture. KI (13.59 g, 81.88 mmol, 1 eq.) was added into the mixture. The mixture was stirred at 95 °C for 5 h. The mixture was cooled to room temperature and diluted with water and EtOAc. The organic layer was washed by brine, dried with Na_2_SO_4_, concentrated and the residue was purified by column chromatography on silica gel eluted with PE/EtOAc (100:0-80:20) and afford 3-hexadecoxypropan-1-ol (12.5 g, 50.80%) as white solid.

B: To a mixture of [rac-(1S)-1-[(4-amino-2-oxo-pyrimidin-1-yl)methyl]-2-hydroxy-ethoxy]methylphosphonic acid (1.5 g, 5.37 mmol, 1 eq.) in DMF (25 mL) was added DIPEA (10 mL).The mixture was stirred at 45 °C for 2 h. The solvent was removed by concentration and the residue was added DMF (25 mL), 3-hexadecoxypropan-1-ol (2.4 g, 8.06 mmol, 1.5 eq.) DIPEA (4.17 g, 32.24 mmol, 5.61 mL, 6.0 eq.) and pyBOP (8.39 g, 16.12 mmol, 3.0 eq.). The mixture was stirred at 45 °C for 16 h. The solvent was removed by concentrated and the residue was purified by column chromatography on silica gel eluted with DCM/MeOH (100:0-90:10) to afford 4-amino-1-[[rac-(5S)-2-(3-hexadecoxypropoxy)-2-oxo-1,4,2dioxaphosphinan-5-yl]methyl]pyrimidin-2-one (2 g, 68.47% ).

C: To a mixure of 4-amino-1-[[rac-(5S)-2-(3-hexadecoxypropoxy)-2-oxo-1,4,2dioxaphosphinan-5-yl]methyl]pyrimidin-2-one (100 mg, 183.93 μmol, 1 eq.) in aq.NaOH (0.5 M, 3.68 mL, 10 eq.) was stirred at room temperature (about 27 °C) for 4 h. The mixture become almost clean. The mixture was cooled to about 0 °C, and then 1 N HCl was added into the mixture until the pH was about 1, and a lot of solid was formed. The solid was collected by filtration and the product was dried under vacuum condition to afford 3-hexadecoxypropoxy-[[rac-(1S)-1-[(4-amino-2-oxo-pyrimidin-1-yl)methyl]-2-hydroxy-ethoxy]methyl]phosphinic acid (50 mg, 47.09%) as off-white solid. ^1^H NMR (500 MHz, CD_3_Cl_3_)δ ppm: 0.84 (t, J=6.6 Hz, 3H), 1.21 (s, 26H), 1.49 (s, 2H), 1.74-1.91 (m, 2H), 3.28-3.86 (m, 10H), 3.93 (d, J=6.4 Hz, 2H), 4.22 (d, J=13.9 Hz, 1H), 6.11 (s, 1H), 7.70 (d, J=7.2 Hz, 1H);m/z (ESI^+^):562.6(M +H).

D: A mixture of 3-hexadecoxypropoxy-[[rac-(1S)-1-[(4-amino-2-oxo-pyrimidin-1-yl)methyl]-2-hydroxy-ethoxy]methyl]phosphinic acid (100 mg, 178.03 μmol, 1 eq.) in HCOOH (6 mL) was heated to 60°C for 16 h. The solvent was removed by concentration. The residue was added MeOH (5 mL), and MeCN (8 mL) solid was formed, and stirred at rt for 30 min. The solid was collected by filtration, then dried under frozen condition to afford 3-hexadecoxypropoxy-[[rac-(1S)-1-[(4-amino-2-oxo-pyrimidin-1-yl)methyl]-2-formyloxy-ethoxy]methyl]phosphinic acid (70 mg, 113.90 μmol, 63.97% yield, 97.86% purity). ^1^H NMR (500 MHz, CD_3_OD)δ ppm:0.89 (t, J=6.8 Hz, 3H), 1.28 (s, 26H), 1.54 (s, 2H), 1.77-1.90 (m, 2H), 3.41 (t, J=6.5 Hz, 2H), 3.50 (t, J=6.4 Hz, 2H), 3.54-3.62 (m, 1H), 3.84 (dd, J=21.9, 14.1 Hz, 2H), 3.92 (d, J=6.0 Hz, 2H), 3.98 (s, 1H), 4.17 (d, J=12.5 Hz, 2H), 4.40 (d, J=7.7 Hz, 1H), 5.96 (d, J=7.4 Hz, 1H), 7.89 (d, J=7.1 Hz, 1H), 8.13 (s, 1H); m/z (ESI^+^):590.5(M + H). ¹H NMR and mass spectra are shown in supplementary Figure 10.

**2. The synthesis scheme and procedure of ODE-(S)-HPMPA formate**

A: To a solution of diethoxyphosphorylmethanol (20 g, 118.96 mmol, 1 eq.) in DCM (400 mL) was added TEA (14.44 g, 142.75 mmol, 19.84 mL, 1.2 eq.). The mixture was cooled to about -60 °C. Then trifluoromethylsulfonyl trifluoromethanesulfonate (38.60 g, 136.80 mmol, 23.02 mL, 1.15 eq.) was added into the mixture at about -60 °C. The mixture was stirred at about -60 °C for 0.5 h, then warm up to room temperature and stirred at room temperature for 1 h. The solvent was removed by concentrated, the residue was diluted with EtOAc (500 mL), washed by water, 1N HCl, followed by brine, dried with Na_2_SO_4_, concentrated to afford diethoxyphosphorylmethyl trifluoromethanesulfonate (28 g, 78.41%) as yellow oil.

B: To a mixture of NaH (4.50 g, 112.48 mmol, 60% purity, 1.5 eq.) in DMF (150 mL) was cooled to about 0 °C. The solution of ethylene glycol (20.94 g, 337.44 mmol, 4.5 eq.) in DMF (100 mL) was added into the mixture. The mixture was stirred at room temperature for 10 min. 1-bromooctadecane (25 g, 74.99 mmol, 1 eq.) was added into the mixture. NaI (11.24 g, 74.99 mmol, 1 eq.) was added into the mixture. The mixture was stirred at 95 °C for 16 h. The mixture was cooled to room temperature and diluted with water and EtOAc. The organic layer was washed by brine, dried with Na_2_SO_4_, concentrated and the residue was recrystallized from PE to afford 2-octadecoxyethanol (11 g, 46.64%) as off-white solid.

C: To a mixture of 9H-purin-6-amine (4.9 g, 36.26 mmol, 1 eq.) in DMF (100 mL) was added rac-(2S)-2-(trityloxymethyl)oxirane (10.33 g, 32.64 mmol, 0.9 eq.). DBU (1.10 g, 7.25 mmol, 1.08 mL, 0.2 eq.) was added into the mixture. The mixture was stirred at 100 °C for 6 h. The solvent was removed by concentration and the residue was purified by column chromatography on silica gel eluted with DCM/MeOH (100:0-94:6) to afford rac-(2S)-1-(6-aminopurin-9-yl)-3-trityloxy-propan-2-ol (7 g, 42.75%) as white solid.

D: To a solution of rac-(2S)-1-(6-aminopurin-9-yl)-3-trityloxy-propan-2-ol (5 g, 11.07 mmol, 1 eq.) in THF (150 mL) was cooled to about -60 °C. LiHMDs (1.0 M, 33.22 mL, 3.0 eq.) was added dropwise into the mixture at about -60 °C (no more than -50 °C). The mixture was stirred at about -60 °C for 15 min, then the solution of diethoxyphosphorylmethyl 2-methylpropane-2-sulfonate (8.30 g, 28.79 mmol, 2.6 eq.) in THF (20 mL) was added dropwise into the mixture (no more than -50 °C). The mixture was warmed up to room temperature and stirred for 16 h. The solvent was removed about 3/5 by concentration and the residue was diluted with EtOAc (300 mL), washed by water, followed by brine. The organic layer was concentrated and the residue was purified by column chromatography on silica gel eluted with DCM/MeOH from 100:0 to 92:8 to afford 9-[rac-(2S)-2-(diethoxyphosphorylmethoxy)-3-trityloxy-propyl]purin-6-amine (3.5 g, 52.53%) as white solid.

E: To a mixture of 9-[rac-(2S)-2-(diethoxyphosphorylmethoxy)-3-trityloxy-propyl]purin-6-amine (3.5 g, 5.82 mmol, 1 eq.) in AcOH (50 mL) and H_2_O (12 mL) was stirred at 90 °C for 3 h. The solvent was removed by concentration and the residue was purified by column chromatography on silica gel eluted with DCM/MeOH (100:0-90:10) to afford rac-(2S)-3-(6-aminopurin-9-yl)-2-(diethoxyphosphorylmethoxy)propan-1-ol (1.6 g, 76.54%) as white solid.

F: To a mixture of rac-(2S)-3-(6-aminopurin-9-yl)-2-(diethoxyphosphorylmethoxy)propan-1-ol (0.8 g, 2.23 mmol, 1 eq.) in MeCN (10 mL) was added bromo(trimethyl)silane (1.36 g, 8.91 mmol, 1.18 mL, 4.0 eq.). The mixture was stirred at room temperature for 16 h. The mixture was concentrated and then diluted with water (10 mL), dried under frozen condition to afford [rac-(1S)-1-[(6-aminopurin-9-yl) methyl]-2-hydroxy-ethoxy]methylphosphonic acid (675 mg, 99.10%).

G: To a mixture of [rac-(1S)-1-[(6-aminopurin-9-yl) methyl]-2-hydroxy-ethoxy]methylphosphonic acid (200 mg, 659.61 μmol, 1 eq.) in DMF (10 mL) was added DIPEA (1 mL). The mixture was stirred at 45 °C for 2 h. The solvent was removed by concentration. The residue was added DMF (10 mL), DIPEA (511.49 mg, 3.96 mmol, 689.33 μL, 6.0 eq.) and pyBOP (1.03 g, 1.98 mmol, 3.0 eq.). The mixture was stirred at 45 °C for 3 h. The mixture become brown and clean. The mixture was stirred 45 °C for 16 h. The mixture was diluted with EA (40 mL), washed by water, followed by brine. The solvent was removed by concentrated and the residue was purified by column chromatography on silica gel eluted with DCM/MeOH (100:0-90:10) to afford 9-[[rac-(5S)-2-(2-octadecoxyethoxy)-2-oxo-1,4,2dioxaphosphinan-5-yl]methyl]purin-6-amine (220 mg, 369.90 μmol, 56.08% yield, 97.81% purity). ^1^H NMR (500 MHz, CDCl_3_) δ ppm: 0.87 (t, J=6.8 Hz, 3H), 1.24 (s, 30H), 1.54 (s, 2H), 3.43 (dt, J=13.6, 6.7 Hz, 2H), 3.54-3.70 (m, 2H), 3.89 (dd, J=13.1, 6.2 Hz, 1H), 4.04-4.66 (m, 8H), 6.02 (d, J=34.8 Hz, 2H),7.87 (d, J=3.0 Hz, 1H), 8.34 (d, J=3.8 Hz, 1H); m/z (ESI^+^):582.5(M +H).

H: To a mixture of 9-[[rac-(5S)-2-(2-octadecoxyethoxy)-2-oxo-1,4,2dioxaphosphinan-5-yl]methyl]purin-6-amine (70 mg, 120.33 μmol, 1 eq.)  in NaOH (0.5 M, 1.20 mL, 5.0 eq.) was added water (1 mL). The mixture was stirred at rt for 2 days. The pH of the mixture was adjusted to about 1 by adding 1 N HCl, and a lot of solid was formed. The solid was collected by concentration. The solid was stirred in MeOH (4 mL) for 10 min and collected by filtration to afford 2-octadecoxyethoxy-[[rac-(1S)-1-[(6-aminopurin-9-yl)methyl]-2-hydroxy-ethoxy]methyl]phosphinic acid (65 mg, 101.75 μmol, 84.56% yield, 93.88% purity). ^1^H NMR (500 MHz, CDCl_3_) δ ppm: 0.84 (t, J=6.8 Hz, 3H), 1.21 (s, 30H), 1.51 (d, J=6.7 Hz, 2H), 3.42 (t, J=7.0 Hz, 2H), 3.49 (d, J=8.9 Hz, 1H), 3.57 (t, J=4.5 Hz, 2H), 3.70 (dd, J=21.7, 8.8 Hz, 2H), 3.84 (d, J=8.7 Hz, 2H), 4.07 (d, J=6.1 Hz, 2H), 4.34 (dd, J=14.2, 7.3 Hz, 1H), 4.47 (d, J=11.7 Hz, 1H), 8.15 (s, 1H), 8.30 (s, 1H); m/z (ESI^+^):600.6(M +H).

I: To a solution of 2-octadecoxyethoxy-[[rac-(1S)-1-[(6-aminopurin-9-yl)methyl]-2-hydroxy-ethoxy]methyl]phosphinic acid (30 mg, 50.02 μmol, 1 eq.) in HCOOH (4 mL) was stirred at 60 °C for 16 h. The solvent was removed by concentration. The residue was added MeOH (5 mL), solid was formed, and stirred at room temperature for 30 min. The solid was collected by filtration, then dried under frozen condition to afford 2-octadecoxyethoxy-[[rac-(1S)-1-[(6-aminopurin-9-yl)methyl]-2-formyloxy-ethoxy]methyl]phosphinic acid (20 mg, 59.88% yield, 95.03% purity). ^1^H NMR (500 MHz, CD_3_OD) δ ppm: 0.88 (d, J=6.9 Hz, 3H), 1.27 (s, 30H), 1.51 (s, 2H), 3.42 (d, J=6.6 Hz, 2H), 3.55 (s, 2H), 3.72 (s, 1H), 3.82-3.90 (m, 1H), 3.97 (s, 2H), 4.13 (d, J=11.4 Hz, 2H), 4.31 (d, J=7.7 Hz, 1H), 4.45 (d, J=8.1 Hz, 1H), 4.60 (d, J=13.5 Hz, 1H), 8.12 (s, 1H), 8.30 (s, 1H), 8.45 (s, 1H); m/z (ESI^+^):628.39(M +H). ¹H NMR and mass spectra are shown in supplementary Figure 10.

**3. The synthesis scheme and procedure of HDP-(S)-HPMPA formate**

A: To a solution of diethoxyphosphorylmethanol (20 g, 118.96 mmol, 1 eq.) in DCM (400 mL) was added TEA (14.44 g, 142.75 mmol, 19.84 mL, 1.2 eq.). The mixture was cooled to about -60 °C. Then trifluoromethylsulfonyl trifluoromethanesulfonate (38.60 g, 136.80 mmol, 23.02 mL, 1.15 eq.) was added into the mixture at about -60 °C. The mixture was stirred at about -60 °C for 0.5 h, then warm up to room temperature and stirred at room temperature for 1 h. The solvent was removed by concentrated, the residue was diluted with EtOAc (500 mL), washed by water, 1N HCl, followed by brine, dried with Na_2_SO_4_, concentrated to afford diethoxyphosphorylmethyl trifluoromethanesulfonate (28 g, 78.41%) as yellow oil.

B: To a mixture of NaH (4.91 g, 122.82 mmol, 60% purity, 1.5 eq.) in DMF (150 mL) was cooled to about 0 °C. The solution of propane-1,3-diol (28.04 g, 368.45 mmol, 26.63 mL, 4.5 eq.) in DMF (150 mL) was added into the mixture. The mixture was stirred at room temperature for 10 min, and then 1-bromohexadecane (25 g, 81.88 mmol, 25.03 mL, 1.0 eq.) was added into the mixture. KI (13.59 g, 81.88 mmol, 1 eq.) was added into the mixture. The mixture was stirred at 95 °C for 5 h. The mixture was cooled to room temperature and diluted with water and EtOAc. The organic layer was washed by brine, dried with Na_2_SO_4_, concentrated and the residue was purified by column chromatography on silica gel eluted with PE/EtOAc (100:0-80:20) and afford 3-hexadecoxypropan-1-ol (12.5 g, 50.80%) as white solid.

C: To a mixture of 9H-purin-6-amine (4.9 g, 36.26 mmol, 1 eq.) in DMF (100 mL) was added rac-(2S)-2-(trityloxymethyl)oxirane (10.33 g, 32.64 mmol, 0.9 eq.). DBU (1.10 g, 7.25 mmol, 1.08 mL, 0.2 eq.) was added into the mixture. The mixture was stirred at 100 °C for 6 h. The solvent was removed by concentration and the residue was purified by column chromatography on silica gel eluted with DCM/MeOH (100:0-94:6) to afford rac-(2S)-1-(6-aminopurin-9-yl)-3-trityloxy-propan-2-ol (7 g, 42.75%) as white solid.

D: To a solution of rac-(2S)-1-(6-aminopurin-9-yl)-3-trityloxy-propan-2-ol (5 g, 11.07 mmol, 1 eq.) in THF (150 mL) was cooled to about -60 °C. LiHMDs (1.0 M, 33.22 mL, 3.0 eq.) was added dropwise into the mixture at about -60 °C (no more than -50 °C). The mixture was stirred at about -60 °C for 15 min, then the solution of diethoxyphosphorylmethyl 2-methylpropane-2-sulfonate (8.30 g, 28.79 mmol, 2.6 eq.) in THF (20 mL) was added dropwise into the mixture (no more than -50 °C). The mixture was warmed up to room temperature and stirred for 16 h. The solvent was removed about 3/5 by concentration and the residue was diluted with EtOAc (300 mL), washed by water, followed by brine. The organic layer was concentrated and the residue was purified by column chromatography on silica gel eluted with DCM/MeOH from 100:0 to 92:8 to afford 9-[rac-(2S)-2-(diethoxyphosphorylmethoxy)-3-trityloxy-propyl]purin-6-amine (3.5 g, 52.53%) as white solid.

E: To a mixture of 9-[rac-(2S)-2-(diethoxyphosphorylmethoxy)-3-trityloxy-propyl]purin-6-amine (3.5 g, 5.82 mmol, 1 eq.) in AcOH (50 mL) and H_2_O (12 mL) was stirred at 90 °C for 3 h. The solvent was removed by concentration and the residue was purified by column chromatography on silica gel eluted with DCM/MeOH (100:0-90:10) to afford rac-(2S)-3-(6-aminopurin-9-yl)-2-(diethoxyphosphorylmethoxy)propan-1-ol (1.6 g, 76.54%) as white solid.

F: To a mixture of rac-(2S)-3-(6-aminopurin-9-yl)-2-(diethoxyphosphorylmethoxy)propan-1-ol (0.8 g, 2.23 mmol, 1 eq.) in MeCN (10 mL) was added bromo(trimethyl)silane (1.36 g, 8.91 mmol, 1.18 mL, 4.0 eq.). The mixture was stirred at room temperature for 16 h. The mixture was concentrated and then diluted with water (10 mL), dried under frozen condition to afford [rac-(1S)-1-[(6-aminopurin-9-yl) methyl]-2-hydroxy-ethoxy]methylphosphonic acid (675 mg, 99.10%).

G: To a mixture of [rac-(1S)-1-[(6-aminopurin-9-yl) methyl]-2-hydroxy-ethoxy]methylphosphonic acid (675 mg, 2.23 mmol, 1 eq) in DMF (25 mL) was added DIPEA (1.73 g, 13.36 mmol, 2.33 mL, 6.0 eq). The mixture was stirred at 40 °C for 2 h. The solvent was removed by concentration and the residue was added DMF (25 mL), pyBOP (3.48 g, 6.68 mmol, 3.0 eq) and DIPEA (1.73 g, 13.36 mmol, 2.33 mL, 6.0 eq). The mixture was stirred at 45 °C for 4 h. The mixture was diluted with EA (150 mL), washed by water, followed by brine. The solvent was removed by concentrated and the residue was purified by column chromatographyon silica gel eluted with DCM/MeOH (100:0-90:10) to afford 9-[[rac-(5S)-2-(3-hexadecoxypropoxy)-2-oxo-1,4,2dioxaphosphinan-5-yl]methyl]purin-6-amine (600 mg, 1.06 mmol, 47.48% yield)

H: To a mixture of 9-[[rac-(5S)-2-(3-hexadecoxypropoxy)-2-oxo-1,4,2dioxaphosphinan-5-yl] methyl]purin-6-amine (250 mg, 440.37 μmol, 1 eq) in H_2_O (2 mL) was added NaOH (0.5 M, 8.81 mL, 10.0 eq). The mixture was stirred at rt for 16 h. The pH of the mixture was adjusted to about 1 by adding 2 N HCl, and a lot of solid was formed. The solid was collected by concentration. The solid was stirred in MeOH (4 mL) for 10 min and collected by filtration to afford3-hexadecoxypropoxy-[[rac-(1S)-1-[(6-aminopurin-9-yl)methyl]-2-hydroxy-ethoxy] methyl]phosphinic acid (200 mg, 341.46 μmol, 77.54% yield).

I: To a solution of 3-hexadecoxypropoxy-[[rac-(1S)-1-[(6-aminopurin-9-yl)methyl]-2-hydroxy-ethoxy]methyl]phosphinic acid (200 mg, 341.46 μmol, 1 eq*.*) in HCOOH (10 mL) was stirred at 60 °C for 16 h. The solvent was removed by concentration. The residue was diluted with MeCN (5 mL) and MeOH (5 mL), a lot of solid was formed, and stirred at room temperature for 10 min. The solid was collected by filtration, and stirred in MeOH (10 mL) for another 30 min. The solid was collected by filtration, dried under vacuum condition to afford 3-hexadecoxypropoxy-[[rac-(1S)-1-[(6-aminopurin-9-yl)methyl]-2-formyloxy-ethoxy]methyl]phosphinic acid (150 mg, 69.87% yield, 95.03% purity) as white solid. ^1^H NMR (500 MHz, CD_3_OD) δ ppm: 0.89 (t, J=6.7 Hz, 3H), 1.27 (s, 26H), 1.51 (d, J=6.8 Hz, 2H), 1.82 (p, J=6.4 Hz, 2H), 3.39 (t, J=6.6 Hz, 2H), 3.48 (t, J=6.3 Hz, 2H), 3.67 (dd, J=13.1, 8.9 Hz, 1H), 3.85 (dd, J=13.2, 8.9 Hz, 1H), 3.92 (q, J=6.4 Hz, 2H), 4.09-4.21 (m, 2H), 4.33 (d, J=7.0 Hz, 1H), 4.46 (dd, J=14.6, 6.3 Hz, 1H), 4.59 (d, J=14.6 Hz, 1H), 8.13 (s, 1H), 8.31 (s, 1H), 8.42 (s, 1H); m/z (ESI^+^):614.72 (M +H). ¹H NMR and mass spectra are shown in supplementary Figure 10.


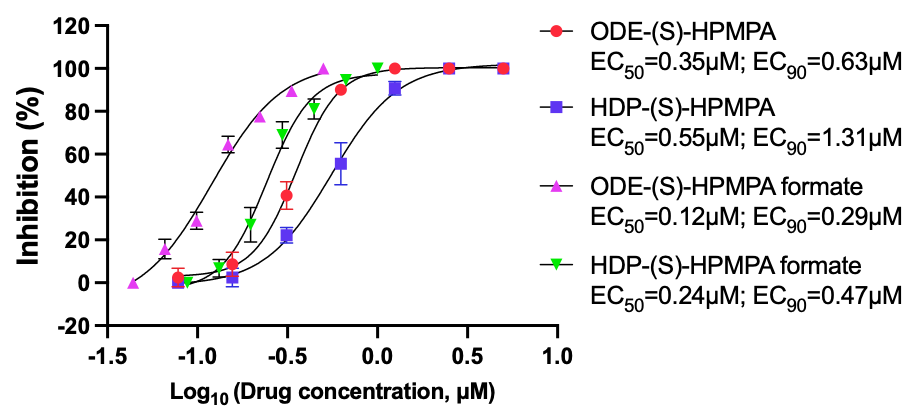


**Figure. S1.**

**In vitro comparisons of anti-vaccinia activities of ODE-(S)-HPMPA formate and HDP-(S)-HPMPA formate to their parent drugs** Vero cells were infected with vaccinia Tiantan strain and treated with the indicated concentrations of ODE-(S)-HPMPA, HDP-(S)-HPMPA, ODE-(S)-HPMPA formate and HDP-(S)-HPMPA formate. All compounds were tested in triplicated wells at each concentration. The solvent was used as the non-treated control. The antiviral effects were determined by calculating the reduction in plaque formation. Data are presented as mean ± SD.


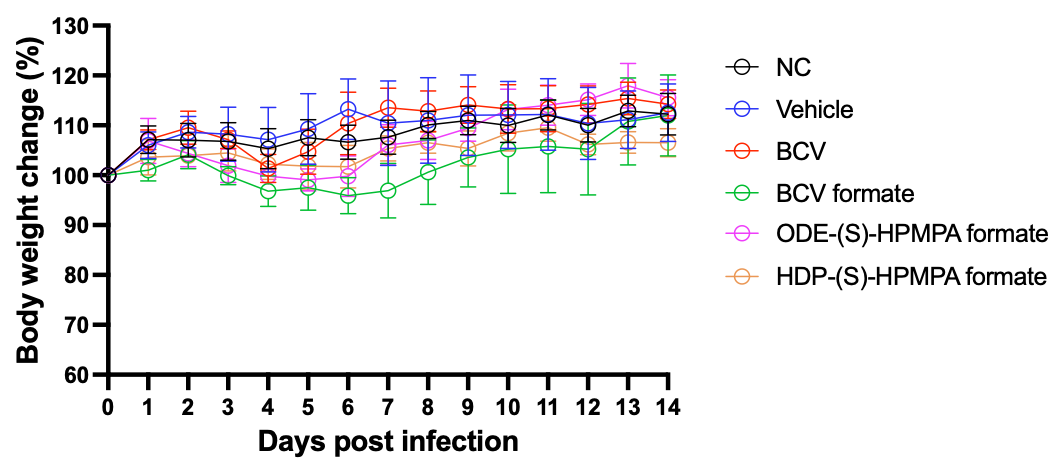


**Figure. S2.**

**Weight loss of uninfected mice treated with each candidate compound** Data are presented as mean ± SD, 5 mice per group.


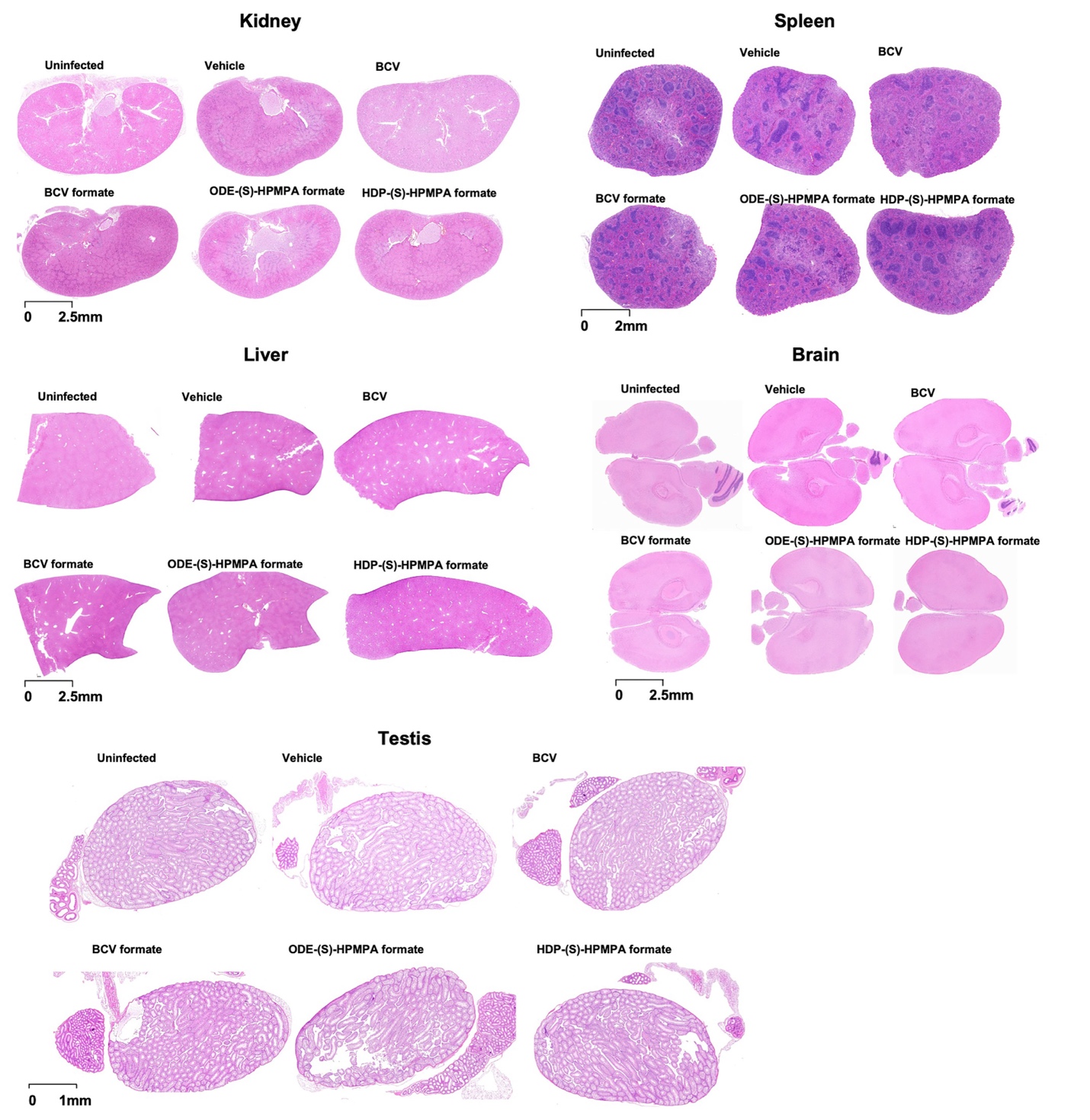


**Figure. S3.**

**No sign of infection-caused or drug-related pathology is detected in key organs post Tiantan vaccinia infection** Histopathological examination of the kidneys, spleen, testis, liver, and brain in mice at 7 days post Tiantan vaccinia infection.

**
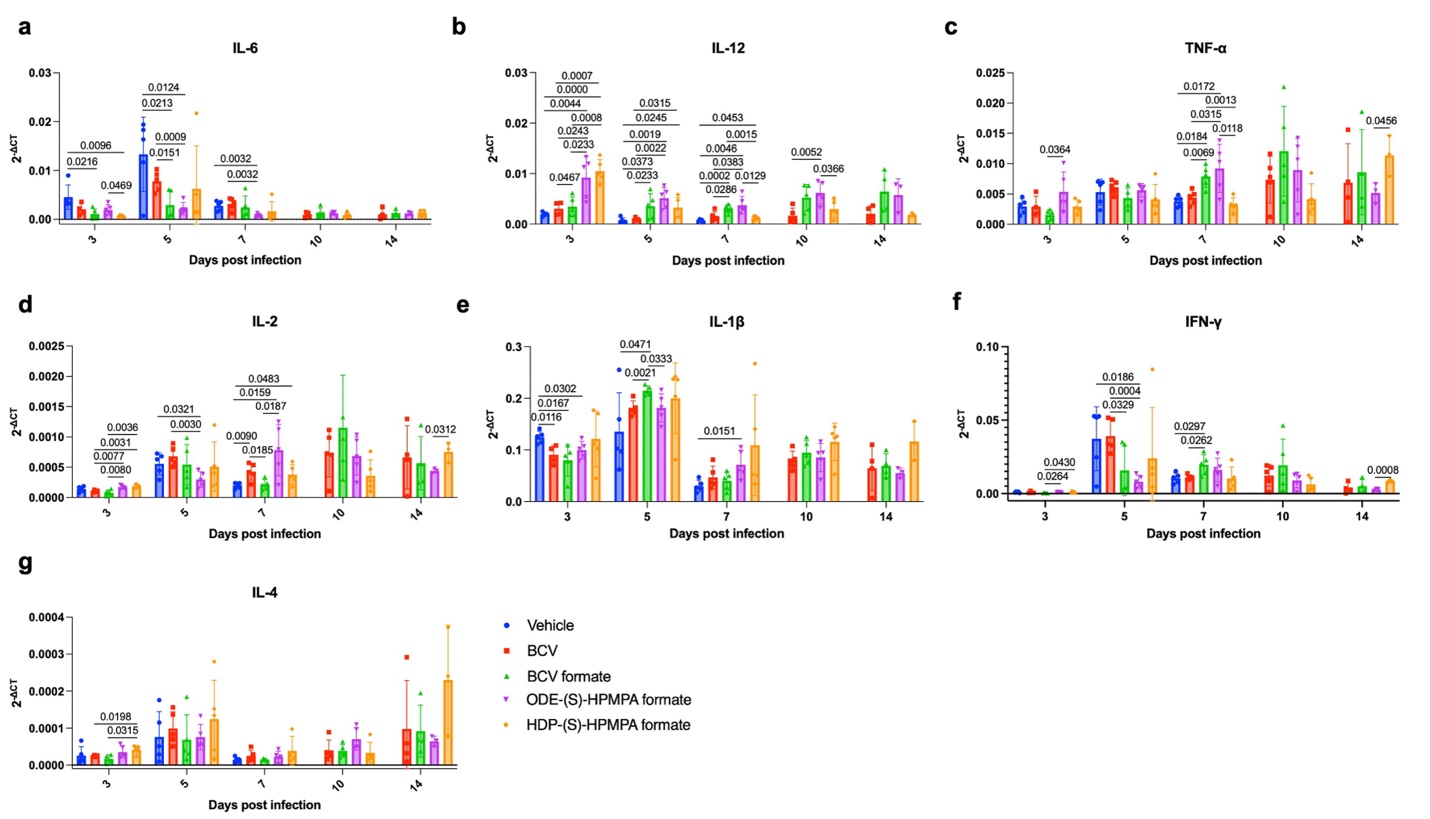
Figure. S4.**

**Drug treatment impacted pulmonary cytokine responses in mice following intranasal infection with Tiantan vaccinia** The transcriptional levels of IL-6 (**a**), IL-12 (**b**), TNF-α (**c**), IL-2 (**d**), IL-1β (**e**), IFN-γ (**f**), IL-4 (**g**) in lung tissues were assessed at 3, 5, 7, 10, and 14 days post-infection with the Tiantan vaccinia virus. Data are presented as mean ± SD.


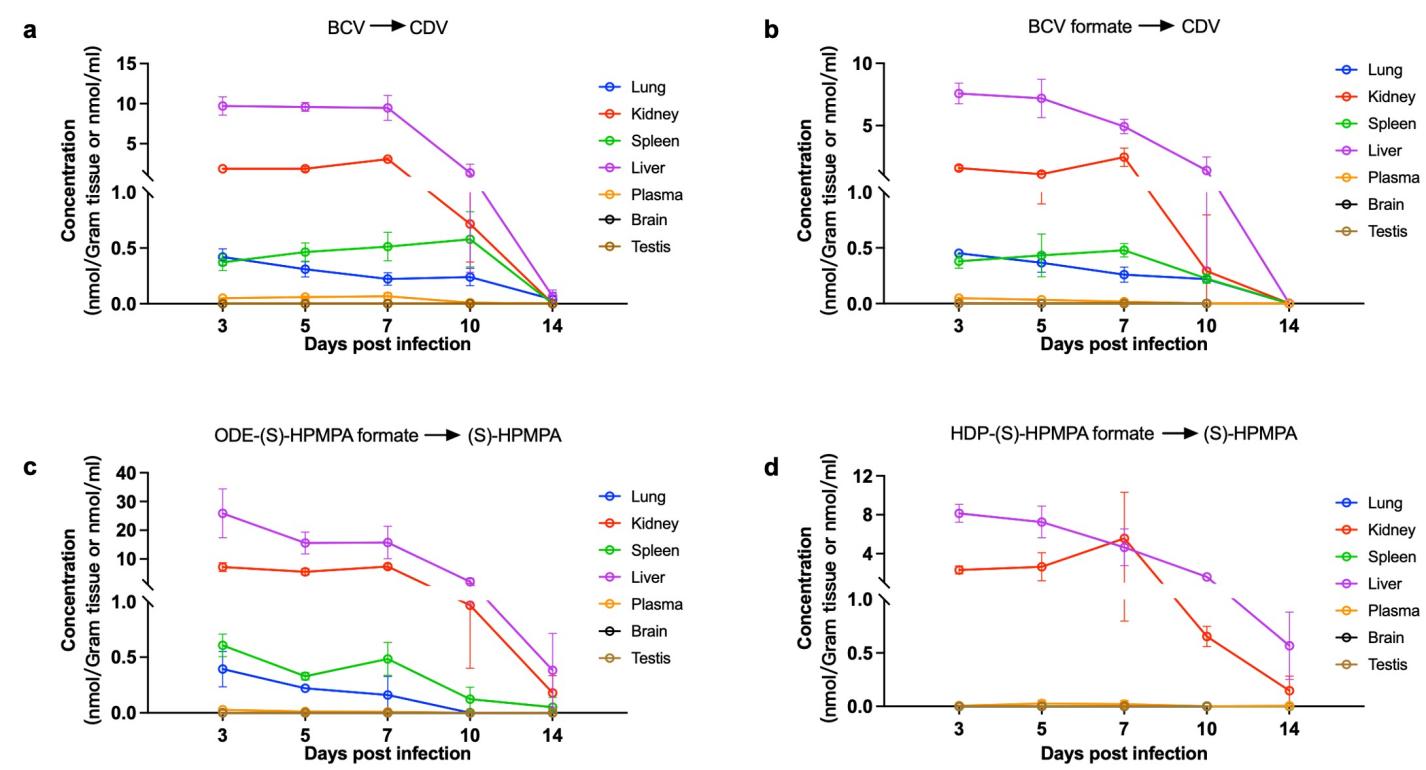
Figure. S5.

**Monitoring in vivo drug metabolites throughout the treatment course (a-d**) The concentrations of drug metabolites in mouse plasma, lung, kidney, spleen and liver were measured using HPLC-MS at 3 days (2 days after the first dose), 5 days (2 days after the second dose), 7 days (2 days after the third dose), 10 days (5 days after the third dose), and 14 days (9 days after the third dose) post infection, respectively (3 mice per group). Metabolites of all drugs were non-detectable in the brain and testis at any time point. Data are presented as mean ± SD.


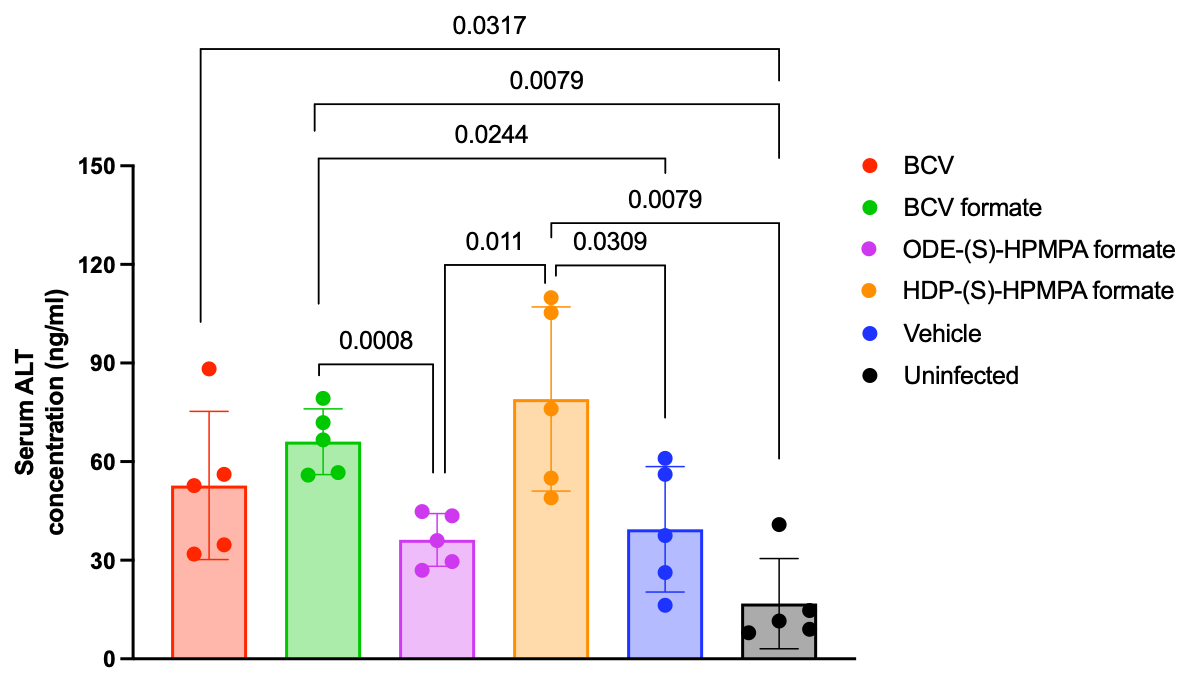


Figure. S6.

**Detection of ALT levels in mouse serum** Serum ALT levels were measured at 2 days after the third dose drug treatment (n=5). Data are presented as mean ± SD.

**
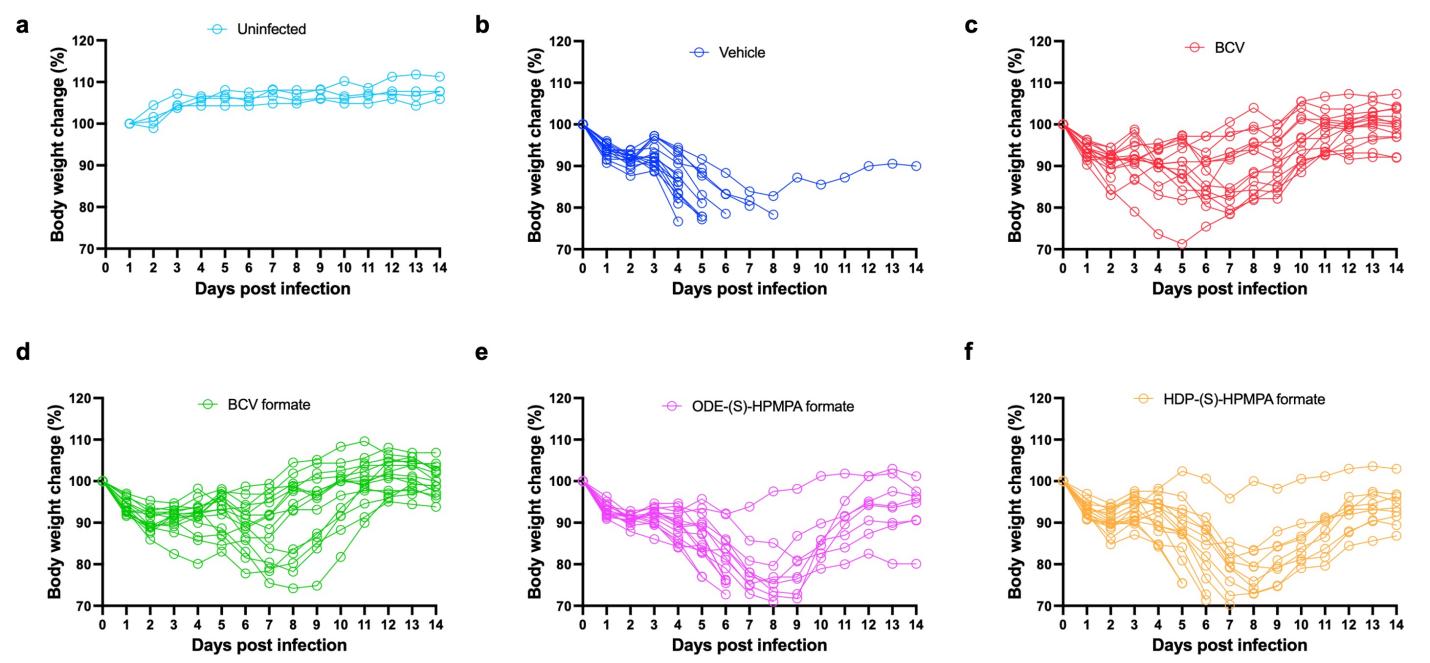
Figure. S7.**

**Weight loss of each individual mouse after being intraperitoneally infected with HSV-1** The mean ± SD of weight changes for each group are presented in Figure 5C.


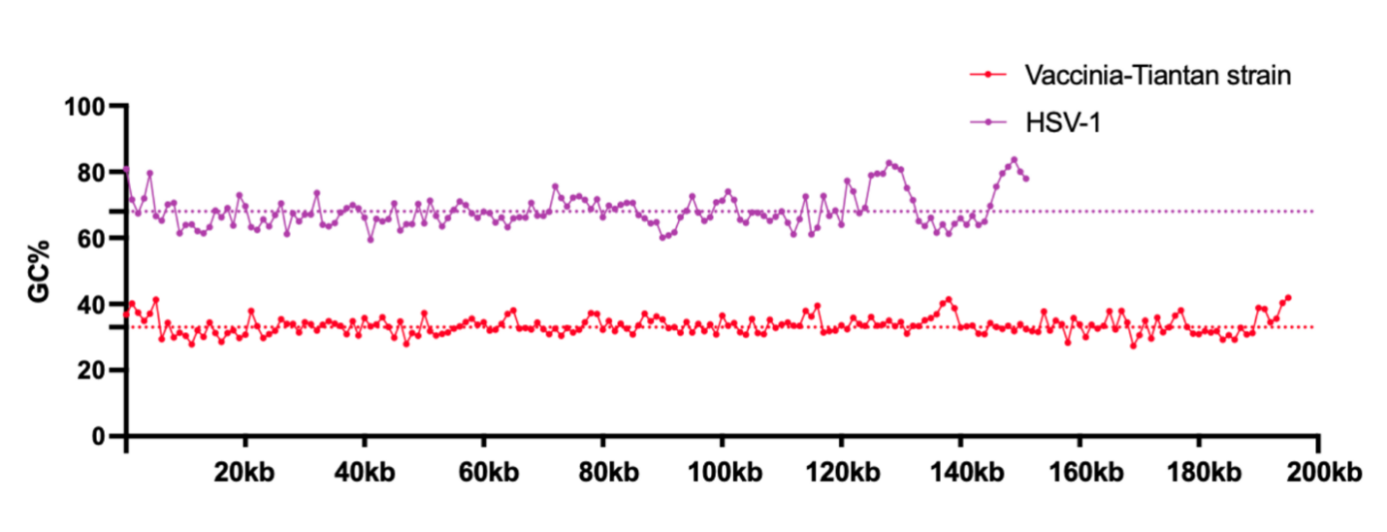
Figure. S8.

**Comparison of genome GC content between Tiantan vaccinia and HSV-1** A representative Tiantan vaccinia genome sequence (accession number: KC207811.1) and a representative HSV-1 genome sequence (accession number: X14112.1) were retrieved from GenBank. The GC content was analyzed using a sliding window approach in the Biostrings package of R package, with a window size of 1000 base pairs.


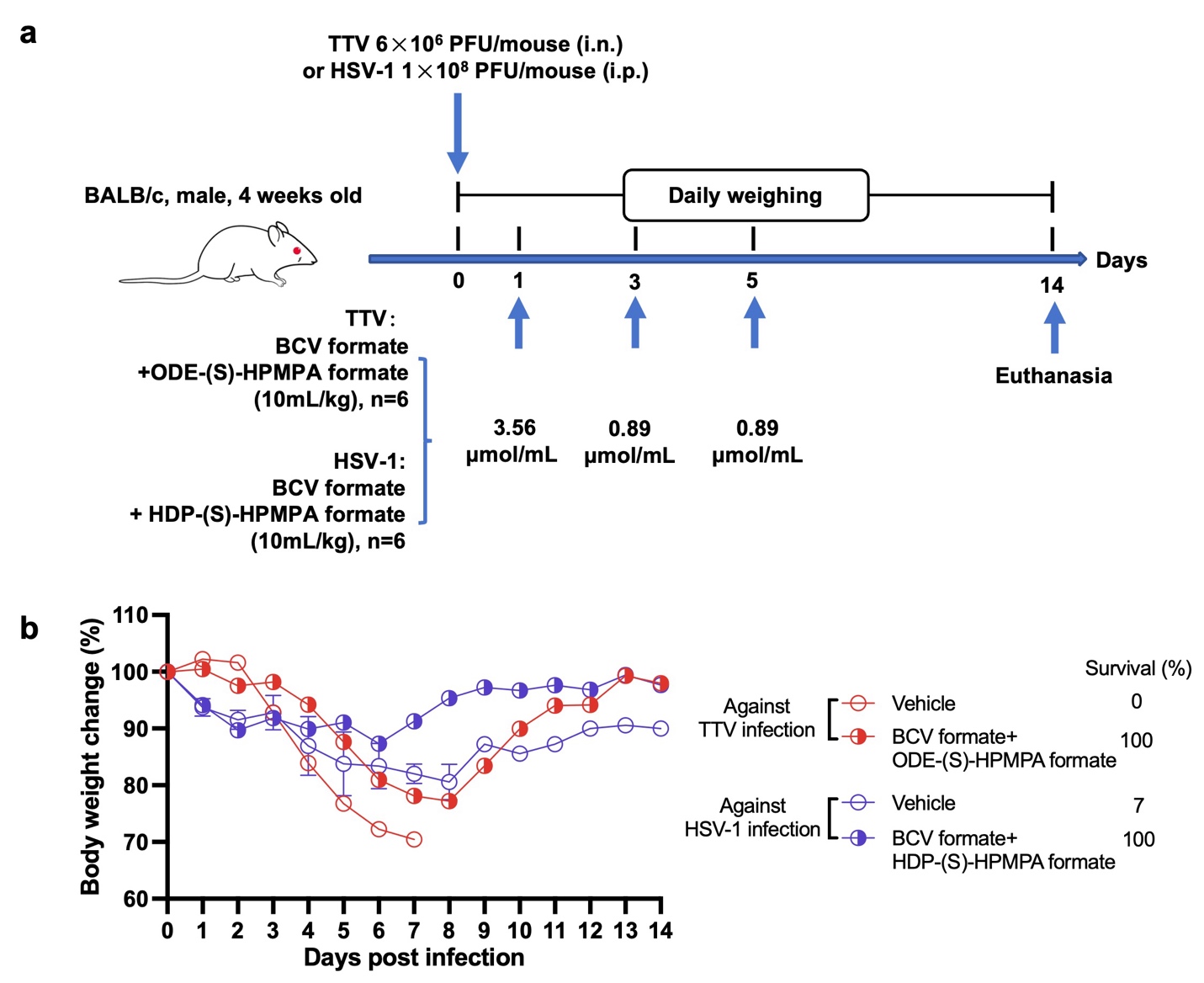


**Figure. S9.**

**Adenine and cytidine analog combinations confer complete protection against both vaccinia and HSV-1 infections** (**a**) The schematic illustration of experimental design. (**b**) Weight loss of mice after being infected with Tiantan vaccinia or HSV-1. The Tiantan vaccinia group shares the same vehicle group as in Figure 3B, and the HSV-1 group shares the same vehicle group as in Figure 5C. Data are presented as mean ± SD.


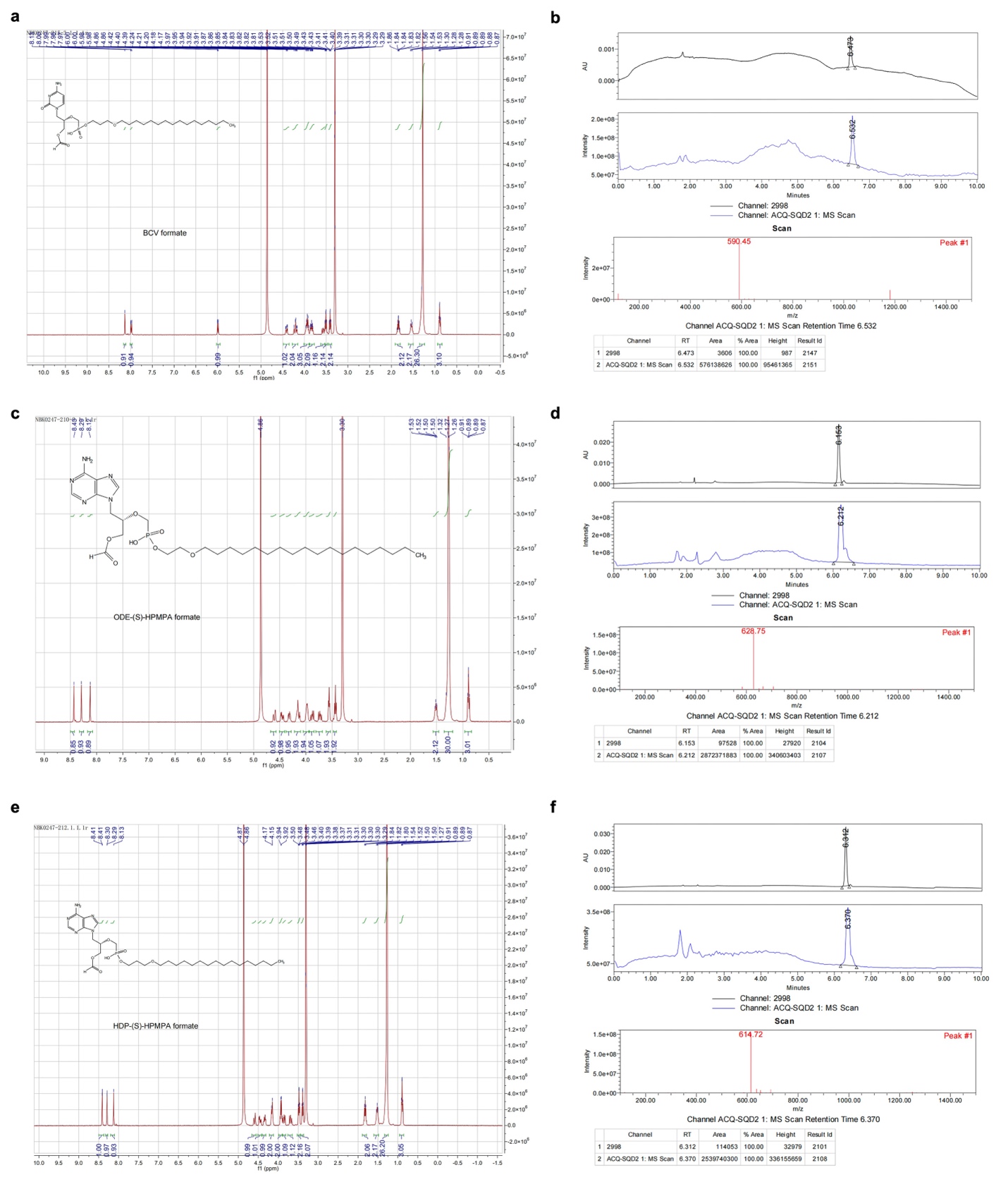
Figure. S10.

**Structural characterization of newly synthesized prodrugs** ¹H NMR and mass spectra of BCV formate (**a-b**), ODE-(S)-HPMPA formate (**c-d**) and HDP-(S)-HPMPA formate (**e-f**).


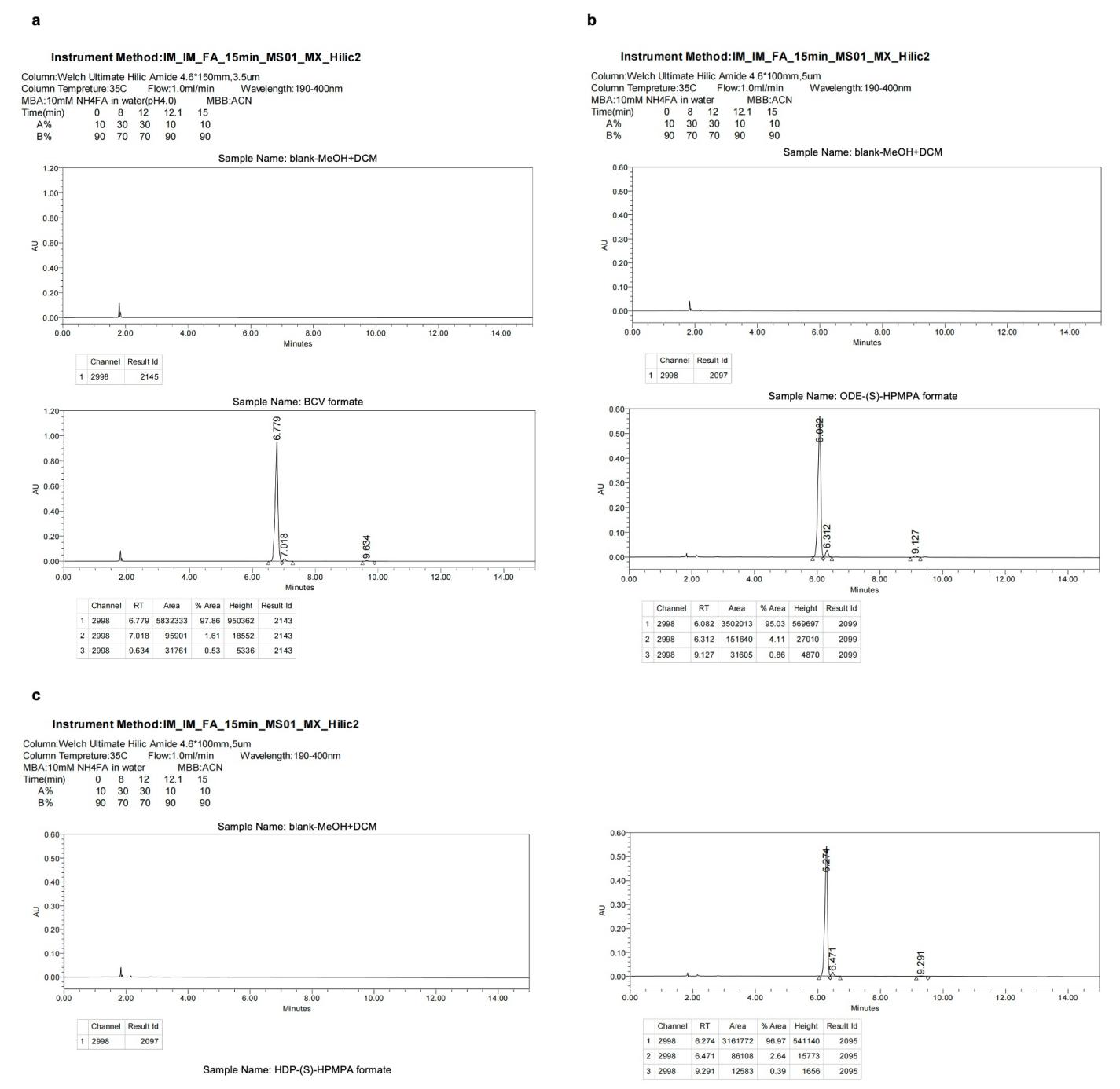
Figure. S11.

The HPLC purity spectra of BCV formate (a), ODE-(S)-HPMPA formate (b) and HDP-(S)-HPMPA formate (c).

**Table S1.** GC content analyses of representative genomes of orthopoxviruses and herpesviruses

| **Virus** | **GenBank Number** | **GC%** |
| --- | --- | --- |
| Vaccinia virus strain TianTan | KC207811.1 | 33 |
| Vaccinia virus WR | AY243312.1 | 33 |
| Monkeypox virus clade IIb B.1 | ON676708.1 | 33 |
| Monkeypox virus clade Ib | PP601207.1 | 33 |
| Monkeypox virus clade Ia | PP601206.1 | 33 |
| Cowpox virus | NC_003663.2 | 33 |
| Variola virus | PP405600.1 | 33 |
| Human herpesvirus 1 | X14112.1 | 68 |
| Human herpesvirus 2 | NC_001798.2 | 70 |
| Human cytomegalovirus | X17403.1 | 57 |
| Epstein-Barr virus | V01555.2 | 60 |

**Table S2.** Both ODE-(S)-HPMPA formate and HDP-(S)-HPMPA formate are more efficacious than ODE-(S)-HPMPA and HDP-(S)-HPMPA in protecting mice from lethal vaccinia infection

| **Compound** | **Treatment schedule and dosage** | **Ratio of biological death** | **Observation period** |
| --- | --- | --- | --- |
| ODE-(S)-HPMPA formate | Oral gavage at day 1 (22.4 mg/kg), days 3 and 5 (5.59 mg/kg) after infection with Tiantan vaccinia ^$^ | 0/11 | 14 days |
| HDP-(S)-HPMPA formate | Oral gavage at day 1 (21.9 mg/kg), days 3 and 5 (5.46 mg/kg) after infection with Tiantan vaccinia ^$^ | 0/12 |  |
| ODE-(S)-HPMPA ^£^ | Oral gavage once daily at 30 mg/kg for 5 days starting 1 day after infection with vaccinia WR strain | 2/15 | 21 days |
| HDP-(S)-HPMPA ^£^ |  | 2/15 |  |

^$^ The mass dose was calculated to ensure the molar doses of the two drugs were equal.

^£^ Values were reported previously ^42^.

**Table S3.** Primer sequences used for quantitative PCR assays of murine cytokines

FP: Forward primer; RP: Reverse primer

| **Gene** |  | | **Primer sequence 5' to 3'** |
| --- | --- | --- | --- |
| GAPDH | | FP | ACCCAGAAGACTGTGGATGG |
|  |  | RP | GGATGCAGGGATGATGTTCT |
| IL6 | | FP | GAGGATACCACTCCCAACAGACC |
|  |  | RP | AAGTGCATCATCGTTGTTCATACA |
| IL-1β | | FP | CAACCAACAAGTGATATTCTCCATG |
|  |  | RP | GATCCACACTCTCCAGCTGCA |
| TNF-α | | FP | CATCTTCTCAAAATTCGAGTGACAA |
|  |  | RP | TGGGAGTAGACAAGGTACAACCC |
| IL-2 | | FP | TCTGCGGCATGTTCTGGATTT |
|  |  | RP | ATGTGTTGTCAGAGCCCTTTAG |
| IFN-γ | | FP | ACAGCAAGGCGAAAAAGGATG |
|  |  | RP | TGGTGGACCACTCGGATGA |
| IL-4 | | FP | ATCGGCATTTTGAACGAGGTC |
|  |  | RP | GAGGACGTTTGGCACATCCA |
| IL-12 | | FP | CTGTGCCTTGGTAGCATCTATG |
|  |  | RP | CGCAGAGTCTCGCCATTATGAT |
| IL-10 | | FP | AAGCCTTATCGGAAATGATCCA |
|  |  | RP | GCTCCACTGCCTTGCTCTTATT |
